# Plant/Bacterial Virus-Based Drug Discovery, Drug Delivery, and Therapeutics

**DOI:** 10.3390/pharmaceutics11050211

**Published:** 2019-05-03

**Authors:** Esen Sokullu, Hoda Soleymani Abyaneh, Marc A. Gauthier

**Affiliations:** Institut National de la Recherche Scientifique (INRS), EMT Research Center, Varennes, QC J3X 1S2, Canada; sokullu@emt.inrs.ca (E.S.); hoda.soleymani@emt.inrs.ca (H.S.A.)

**Keywords:** virus, plant, bacteriophage, phage display, drug discovery, encapsulation, drug delivery

## Abstract

Viruses have recently emerged as promising nanomaterials for biotechnological applications. One of the most important applications of viruses is phage display, which has already been employed to identify a broad range of potential therapeutic peptides and antibodies, as well as other biotechnologically relevant polypeptides (including protease inhibitors, minimizing proteins, and cell/organ targeting peptides). Additionally, their high stability, easily modifiable surface, and enormous diversity in shape and size, distinguish viruses from synthetic nanocarriers used for drug delivery. Indeed, several plant and bacterial viruses (e.g., phages) have been investigated and applied as drug carriers. The ability to remove the genetic material within the capsids of some plant viruses and phages produces empty viral-like particles that are replication-deficient and can be loaded with therapeutic agents. This review summarizes the current applications of plant viruses and phages in drug discovery and as drug delivery systems and includes a discussion of the present status of virus-based materials in clinical research, alongside the observed challenges and opportunities.

## 1. Introduction

In the 18th century, the term “virus” was defined as a morbid principle or poisonous substance produced in the body as the result of some disease [1]. This was due to the initial identification of viruses as infectious agents that could be transferred to other humans or animals, similarly to bacteria but of different size. Since the discovery of the first virus by Ivanovsky in 1892, our understanding of the properties of viruses has changed significantly [2]. Owing to advances in virology, their definition was changed to small, non-cellular obligate parasites carrying non-host genetic information [3]. In fact, these advances not only improved our knowledge about the nature of viruses, but also contributed to change their negative connotation. After the discovery of bacterial viruses (bacteriophages; phages), the French Canadian Felix d’Herelle recognized their ability to replicate exponentially and kill bacteria [4]. These observations suggested potential clinical applications for bacteriophages such as their use as antibacterial agents for the treatment of infectious disease. Although many large pharmaceutical companies marketed phage products in the 1920s and 1930s, clinical failures and theoretical concerns led to their abandonment [5]. In parallel, the discovery of antibiotics equally contributed to the loss of interest in phage therapy around this time [6]. Nonetheless, in the 1940s bacteriophages regained attention in the field of molecular biology. They were notably used as model organisms to understand the genetic basis of virus–host interactions in addition to enabling the discovery of several genetic processes such as transcription, translation, recombination, and regulation of gene expression [7]. While these discoveries reshaped the paradigm of virology, in 1985 the invention of the so-called “phage display” technique greatly broadened the field to new areas of application including drug discovery, vaccine development, antibody engineering, enzyme evolution, and gene therapy [8,9]. Thirty-three years after its development, the contribution of phage display technology to the selection of peptides and antibodies was recognized by a Nobel Prize in Chemistry in 2018 [10]. Another milestone was reached around the year 2000, when viruses were recognized as nanomaterials with the ability to encapsulate molecules within their capsids, to template biomineralization of inorganic materials, and to form self-assembled 2D/3D nanostructures. In this regard, viruses have become promising materials for several applications ranging from biosensing, imaging, and targeted drug/gene delivery to energy/electronic applications (memory devices, batteries, and light-harvesting systems). This review summarizes the current applications of plant and bacterial viruses in medicine, with a focus on virus-based drug discovery approaches and drug delivery systems. Human/animal viruses used e.g., for gene delivery, have been reviewed elsewhere and will not be discussed herein [11,12]. The review begins with an overview of the structure and chemistry of the most common plant and bacterial viruses used in nanomedicine and is concluded by a discussion of the present status of virus-based materials in clinical research, alongside current existing challenges and opportunities.

## 2. Viruses as Nanomedicine

From a material point of view, viruses can be considered as protein-based supramolecular assemblies composed of multiple copies of coat proteins assembled into shell structures of different shapes/sizes ranging from tens to hundreds of nanometers. The protein outer shell (i.e., the so-called capsid) encapsulates the genomic material that contains all essential genes to replicate within a host [13]. The primary function of the capsid is to protect the genomic material and this feature makes viruses stable under conditions such as extreme temperature and pH [14]. Although animal viruses are widely recognized as a delivery vehicle, or ‘vector’, for gene therapy, their use as a nanocarrier has remained relatively limited due to safety concerns. Indeed, while it has been demonstrated that the administration of 10^11^ plant viruses or phage to mice showed no sign of toxicity, the same dose of animal viruses caused severe hepatotoxicity [15,16,17,18]. Therefore, plant and bacteria viruses have received considerably more attention than animal viruses in nanomedicine for applications other than gene delivery. The most studied bacterial virus is M13 phage, whereas the tobacco mosaic virus (TMV), cowpea chlorotic mottle virus (CCMV), and cowpea mosaic virus (CMV) are the most extensively studied plant viruses [19]. Viruses possess precise, nanoscale structures and dimensions that are difficult replicate using chemical synthesis or top-down fabrication methods [20]. The diversity in shape and size of viruses provides a wealth of possibilities to researchers, who can choose the most appropriate system for a given application. For instance, while viruses with higher aspect ratios are more suitable to target diseased vessel walls, viruses with flexible rod shapes have been shown to penetrate better into tumor tissue [21,22]. In addition, the surface properties of viruses can be controlled using chemical and genetic approaches without destroying their structural integrity. This feature enables spatial control on the position of functional moieties, such as targeting ligands, drug molecules, and contrast agents on the virus surface and allows for the design of multifunctional systems bearing combinations of the above [23]. One of the most interesting properties of viruses, in terms of material synthesis, is that they can be produced in large quantities by infecting host cells, and can be purified inexpensively on a large scale. Therefore, virus-based materials are a niche nanomaterial with several unique features compared to synthetic nanomaterials.

### 2.1. Bacterial Viruses (Bacteriophages)

#### 2.1.1. Filamentous Bacteriophages (M13 and fd)

**Figure 1:**
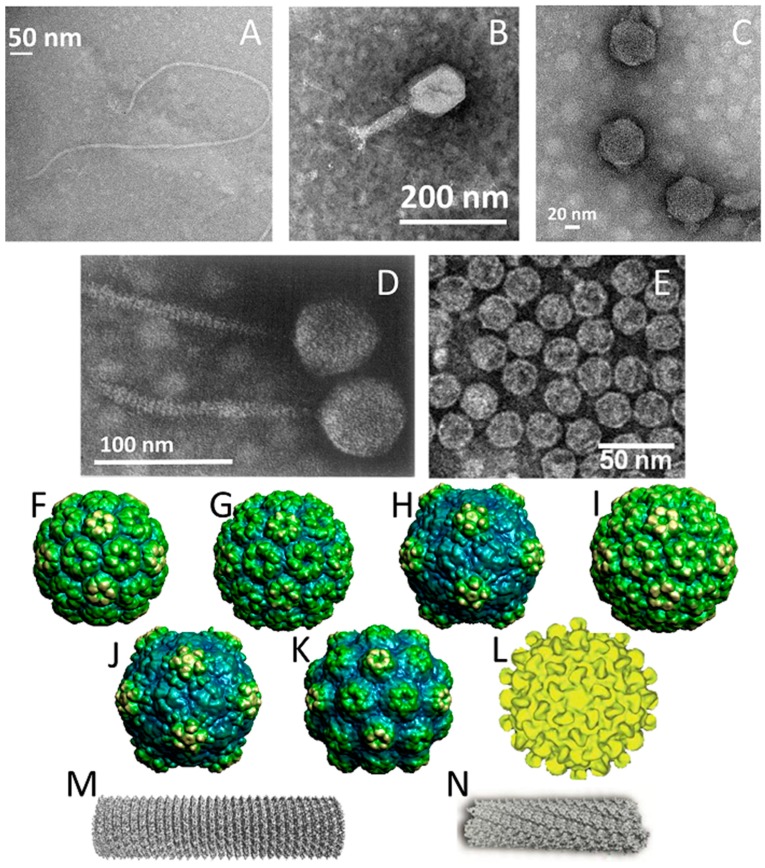
Structures of the viruses discussed in this review. Transmission electron microscopy (TEM) images of (**A**) M13 phage, (**B**) T4 phage, (**C**) T7 phage, (**D**) λ (lambda) phage, and (**E**) MS2 phage. (TEM Images were acquired by the authors, except for λ phage (reprinted with permission from [36], Copyright Elsevier, 1968) and TEM image of MS2 phage (reprinted with permission from [37], Copyright The Royal Society of Chemistry, conveyed through Copyright Clearance Center, Inc., 2011). Structures of plant viruses (**F**) brome mosaic virus (BMV), (**G**) cowpea chlorotic mottle virus (CCMV), (**H**) cowpea mosaic virus (CPMV), (**I**) cucumber mosaic virus (CMV), (**J**) red clover necrotic mosaic virus (RCNMV), (**K**) turnip yellow mosaic virus (TYMV), (**L**) hibiscus chlorotic ringspot virus (HCRSV), (**M**) tobacco mosaic virus (TMV), and (**N**) PVX. (Images of the following viruses were obtained from the VIPERdb (http://viperdb.scripps.edu/) [38]: BMV, CCMV, CPMV, CMV, RCNMV, TYMV. The image of HCRSV was reprinted with permission from [39], Copyright Elsevier, 2003. The image of TMV was reprinted with permission from [40], Copyright Elsevier, 2007. The image of PVX was reprinted with permission from [41], Copyright Elsevier, 2017).

**Figure 2:**
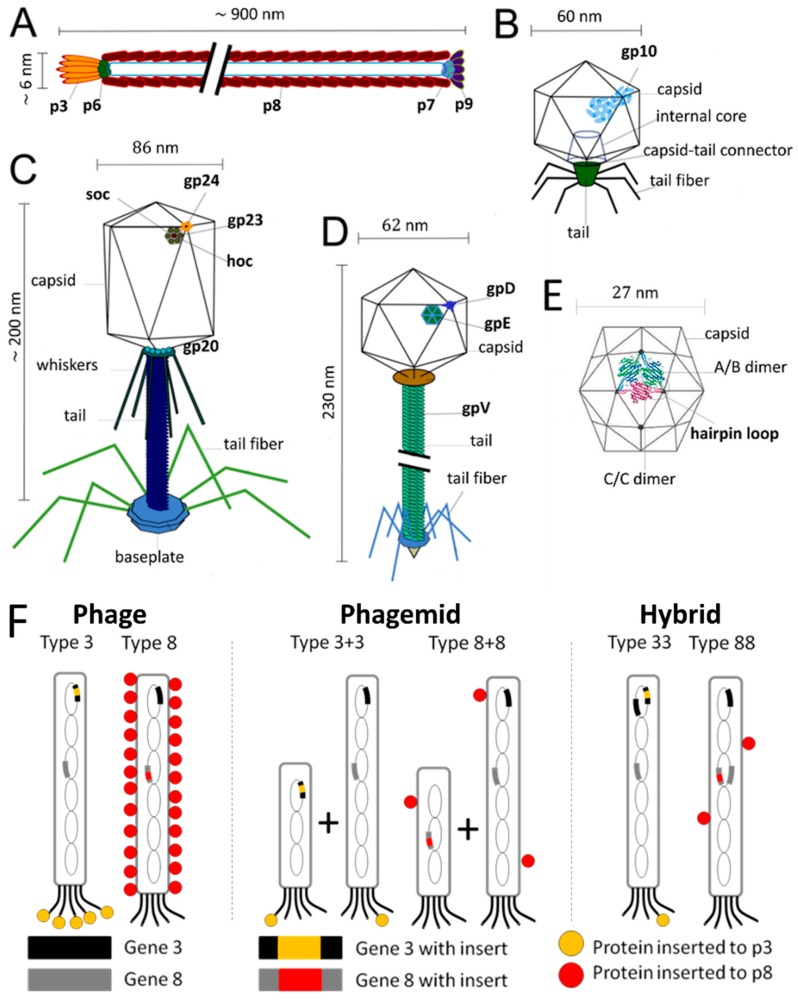
Assembly of coat proteins on bacteriophage (**A**) M13, (**B**) T7, (**C**) T4, (**D**) λ (lambda), and (**E**) MS2 (Images of M13, T7, T4, and λ (lambda) phages were adapted with permission from [89], Copyright American Chemical Society, 2015. The image of MS2 phage was adapted with permission from [90], Copyright the PCCP Owner Societies, 2010). (**F**) Schematic of M13 phage display systems; phage system (type 3/8), phagemid system (type 3+3/8+8), and hybrid system (type 33/88) (The image was adapted with permission from [88], Copyright Elsevier, 1993).

M13 and fd phages belong to a group of filamentous phages (Ff) that specifically infect *Escherichia coli* bacteria. As their genomes are more than 98% identical and their gene products are interchangeable, they are usually collectively referred to as Ff phage [24]. Thus, only the properties of M13 phage are discussed herein as a representative example of filamentous phages. The relatively simple structure of the M13 virion has been extensively studied and is very well known. M13 is 65 Å in diameter and its length depends on the size of enclosed genome (9300 Å in the case of the wild-type M13) (Figure 1A). The flexible filamentous structure contains a circular, 6407 base-pair single-stranded DNA genome coated with 2700 copies of the major coat protein p8 (Figure 2A). The major coat proteins form a tube around the DNA, in an overlapping helical array. The N-terminus of the p8 protein extends towards the exterior of the capsid while the C-terminus interacts with the DNA inside. The hydrophobic domain located in the central part of p8 protein stabilizes the viral particle by interlocking the coat proteins with their neighbors. Additionally, four other minor coat proteins are present, at five copies per particle. p7 and p9 are located at one end of the capsid, while p3 and p6 are located at the other end. p3 is the largest and most complex coat protein and is responsible for the host cell recognition and infection [25,26,27].

M13 phage engages in a chronic infection life cycle where the propagated phage particles are slowly released from the host cell by secretion through the outer membrane, a process that does not lead to bacteria lysis. Phage infection starts with the attachment of p3 protein to the F pilus of bacteria. The phage genome enters the cell and is converted into double-stranded DNA. Afterwards, the synthesis of all M13 phage proteins starts, and the double-stranded DNA is amplified in a process involving p2 and p10 proteins to produce plus-strand copies of the phage DNA. Protein p5 is employed in coating the amplified DNA molecules while the coat proteins p8, p7, p9, p6, and p3 are inserted into the inner bacterial membrane. A small uncovered hairpin of single-stranded DNA is captured by a complex of integral membrane proteins p1, p4, and p9. This complex is described as a membrane pore where the phage is assembled and extruded from the bacterium. As the release of mature M13 virions occurs right after phage assembly, they do not accumulate inside the bacteria and the infected cell continue to grow, albeit at a reduced rate [26,28,29,30,31].

#### 2.1.2. T4 Bacteriophage

The T4 phage is a double-stranded DNA virus that is known as one of the largest viruses to infect bacteria. It belongs to the Myoviridae family and infects *Escherichia coli* and the closely related *Shigella*. Like other members of Myoviridae family, T4 has a prolate icosahedral head, a collar with whiskers, and a contractile tail terminating in a baseplate that is attached to six long tail fibers (Figure 1B). While the fibers recognize the host cell surface and attach to the bacterium during infection, the baseplate binds to specific surface receptors and degrades the bacterial wall with its enzymes. This process enables the introduction of DNA into the cell. The virion consists of several components including DNA, proteins, and a few non-protein constituents such as polyamines associated with DNA (putrescine, spermidine, cadaverine), ATP and Ca^2+^ associated with the tail sheath, and dihydropteroylhexaglutamate associated with the baseplate [32].

The DNA of T4 phage is tightly packed inside the protein capsid, which has a length of 120 nm and a width of 86 nm. The capsid is built from three essential proteins: the major capsid protein gp23* (49 kDa, *: final form within capsid, following enzymatic processing) present at 930 copies, the vertex protein gp24* (47 kDa) present at 55 copies, and the portal protein gp20 (61 kDa) present at 12 copies (Figure 2C). Additionally, there are two outer capsid proteins that are nonessential and bind to the capsid after assembly. The highly antigenic outer capsid protein (Hoc, 39.1 kDa) occupies the center of the gp23 capsomers and is present in up to 155 copies per capsid particle. The rod-like small outer capsid protein (Soc, 9.7 kDa) binds to the capsid surface between the gp23* capsomers (up to 810 copies per capsid) and form a nearly continuous mesh on the surface encircling the gp23* hexamers. Interaction of a Soc protein with two gp23* proteins of adjacent capsomers, as well as trimerization of Soc proteins through C-terminal interactions, stabilize the gp23* hexameric capsomers. Although Soc protein is not essential, the assembly of Soc proteins on the surface of T4 improves stability towards pH (up to pH 11), temperature (<60 °C), osmotic shock, and denaturants. Nevertheless, deletion of either one or both Hoc and Soc genes does not affect phage viability or infectivity under standard laboratory conditions [33,34,35].

#### 2.1.3. T7 Bacteriophage

T7 bacteriophage belongs to the genus of T7-like bacteriophages, which are characterized by their isometric capsid and non-contractile tail (Figure 1C). T7 phage contains a short tail and a 60 nm symmetrical polyhedral capsid with a conspicuous core (composed of proteins gp14, 15, and 16) containing a 40,000 base-pair double-stranded DNA. Phage assembly begins with the formation of a prohead composed of the major head protein gp10A (36.4 kDa), the minor head protein gp10B (41.7 kDa, derived from a read-through of gp10A), and the scaffolding gp9 protein (Figure 2B). During the process of DNA packaging, the prohead interacts with DNA through the terminase proteins (gp18 and 19) and loses the scaffolding protein once the encapsidation of DNA is complete. Afterwards, the connector protein (gp8) that attaches the core to the tail is incorporated into the capsid structure. The function of core structure is believed to be essential for infectivity, but not for the stability of the prohead structure [42,43].

#### 2.1.4. λ (lambda) Phage

λ phage is a temperate *Escherichia coli* virus composed of a flexible helical tail and a 62 nm diameter icosahedral capsid containing a 48,500 base-pair double-stranded DNA genome (Figure 1D) [44]. As a part of its temperate life cycle, λ phage initially integrates its DNA into the bacterial genome where it is replicated with bacterial chromosomes and transmitted to new cells. Thereafter, the lytic cycle begins and phage proteins are synthesized to form the phage particles. The lytic cycle takes ~40 min and produces ~100 phage, and the ends with cell lysis, which releases the phages. The generation of empty procapsids is the first step of phage assembly. Four hundred and five copies of protein E (gpE), one of the major capsid proteins, are organized into hexameric and pentameric capsomers (Figure 2D). The phage DNA is then packed into the procapsid, which provokes a reconfiguration of gpE as well as the expansion of the procapsid. The expansion is followed by the attachment of protein gpD, the head decoration protein, to form mature capsid. Four hundred and twenty copies of protein gpD are present within the capsid and stabilize the expanded capsid structure to prevent DNA release [44,45].

#### 2.1.5. MS2

MS2 is an RNA-containing *Escherichia coli* bacteriophage with a 27 nm diameter icosahedral capsid (Figure 1E). The phage capsid is composed of 180 copies of coat protein and a single copy of maturation protein (A protein) that is responsible for attachment to the host bacterial cell during infection [46]. During the assembly of phage particles, coat proteins initially form dimers and attachment of the dimer to an RNA hairpin produces a complex initiating the growth of the capsid [47]. As the complete RNA sequence is not necessary for initiation of the capsid formation, the self-assembly of purified coat proteins can be achieved with only the RNA hairpin loop to form empty virus-like particles [46,48]. Empty capsids can be also produced by removing the RNA genome in alkaline conditions (pH ~11.8), conditions that are suspected to degrade the RNA molecule through phosphate hydrolysis and reduce its affinity for the capsid proteins. In addition to the ability to produce phage capsids without genetic material, the presence of 32 pores, each with a 1.8 nm diameter, on the capsid surface enables the use this virus as a nanocarrier [49].

### 2.2. Plant Viruses

Like bacteriophages, plant viruses also possess many shapes, sizes, and surface properties, which offer a great diversity of possibilities for medical applications. Plant viruses can be conveniently produced from infected leaves, where infection is achieved by exposure to purified virus particles, infected leaf samples, or simple genomic products of the virus such as cDNA and RNA transcripts [50]. Moreover, plant viruses demonstrate remarkable stability over a wide pH range (3.5–9), temperatures up to 90 °C, and towards a variety of organic solvents (e.g., ethanol, dimethyl sulfoxide) [51,52,53,54]. As the shape of the viruses can significantly affect their performance in a given biomedical application, in the following sections they are categorized based on their shape. There are two main groups of plant viruses: icosahedral viruses with spherical shapes and rod-shaped viruses with high aspect ratios (Table 1).

#### 2.2.1. Icosahedral Plant Viruses

The icosahedral structure is the most common shape for plant viruses. The members of this group most commonly used in medicine are cowpea mosaic virus (CPMV, Figure 1H), cowpea chlorotic mottle virus (CCMV, Figure 1G), brome mosaic virus (BMV, Figure 1F), cucumber mosaic virus (CMV, Figure 1I), hibiscus chlorotic ringspot virus (HCRSV, Figure 1L), red clover necrotic mosaic virus (RCNMV, Figure 1J), and turnip yellow mosaic virus (TYMV, Figure 1K). They are composed of 180 copies of coat protein that form capsid structures with sizes ranging from 28–36 nm (Table 1), each containing a single-stranded RNA genome [51,55,60,61,62,68]. In addition, RNA-free capsids can be generated artificially using pressure, basic/acidic environments, denaturing agents (ca. urea), ribonucleases, or repeated freeze–thaw cycles [51,60,61,68]. In the case of CPMV particles, empty capsids are efficiently produced by co-expressing the fused small/large subunits of coat protein (VP60) along with the 24K proteinase in insect cells and plants [69].

#### 2.2.2. Rod-Shaped Plant Viruses

Tobacco mosaic virus (TMV, Figure 1M) and potato virus x (PVX, Figure 1N) are the rod shaped plant viruses that have been most widely used for drug delivery systems. While both viruses contain single stranded RNA genetic material, they are formed of different coat proteins with different copy numbers, resulting in different lengths. TMV consists of 2130 copies of coat proteins, helically arranged around RNA and forming a hollow nanorod with a 4 nm wide interior channel that is 300 nm in length. It is also possible to produce them as disks in the absence of RNA, which is another nanostructure consisting of 34 coat proteins. The most interesting feature of TMV particles is their ability to form RNA-free spherical nanoparticles from the rod-shaped virus due to thermal processing. The size of the spherical particles can be tuned between 100–800 nm [70,71,72]. On their side, PVX particles have a rod-like shape with sizes similar to that of TMV, and are 515 × 13 nm in size [67,73].

## 3. Virus-Based Drug Discovery

### 3.1. Phage Display Platforms

Various types of phage have been employed to create phage display platforms and have been used for the purpose of drug discovery. This section will present the properties of the most popular of these, with emphasis on their relative advantages and shortcomings.

#### 3.1.1. M13 Phage Display Platform

M13 has been the most widely used phage for phage display since the invention of this technology in 1985 by George P. Smith [74,75]. Combinatorial libraries of polypeptides fused to coat proteins have been used to screen interaction partners towards several targets as well as to study structure–function relations in proteins [25]. In phage display, the genome of M13 phage is manipulated by inserting a DNA sequence into the gene encoding a coat protein. Generally, diverse combinatorial libraries of short peptide sequences (8–12 amino acids) are displayed as fusions to these. Any modification of the phage genome is reflected in a corresponding modification in the coat proteins of the phage, which provides a link between the phenotype and genotype. Selection of the best peptide binding sequence for a given target material is performed through an enrichment process called “biopanning”. Initially, the phage are allowed to bind to the target then, after washing away the non-bound phage, the bound phage are eluted and amplified through host bacterial infection. This artificial evolutionary process to select the best binding peptide sequence is repeated several times for enrichment of the best binding partners. Finally, the selected binding peptides are identified by DNA sequencing of the phage genome [76,77,78]. Phage display technology has been extensively used to identify specific binding peptides for many biological molecules including toxins, bacteria, organs, and tumor-associated antigens [79,80,81,82]. Although all five coat proteins have been used to display foreign proteins, the most common approach is to fuse foreign sequences to the N-terminus of p3 and p8 coat proteins [83,84,85].

There are three different strategies to display proteins as fusions to p3 and p8 coat proteins, which are categorized as phage, phagemid, and hybrid systems. In the phage system, the gene encoding the foreign protein is directly inserted into the phage genome and results in fusion proteins displayed on every copy of chosen coat protein. As a general rule, larger proteins are more efficiently displayed on p3, and the p8 protein is limited to displaying short peptides (~6–10 amino acids). Nonetheless, p3 remains limited in what it can display, and proteins larger than 50 amino acids cannot be displayed on all five copies. Thus, it can be necessary to decrease the copy number of fusion proteins to efficiently display them on the desired coat protein. The phagemid system is used to overcome this limitation. A phagemid is a plasmid carrying the viral gene encoding the fusion coat protein, phage origin of replication, and a phage-packaging signal. The genes required for phage assembly, including the wild type coat protein, are provided by packaging-defective ‘helper’ phage. Upon coinfection of bacteria by phagemid and helper phage, wild type proteins and fusion coat proteins are synthesized and preferentially-assembled around the phagemid DNA. This results in hybrid phages displaying only a few copies of the fusion coat protein. The hybrid system was also invented with a similar motivation to the phagemid system: to enable the display of large protein sequences on the phage’s surface. However, unlike the phagemid system, it only employs the phage genome, which carries both the gene encoding wild type coat protein and the gene encoding fusion protein. Smith defined these three systems using the terms“3”, “3+3”, and “33” respectively (Figure 2F). Number “3” indicates p3 coat protein whereas formats “8”, “8+8”, and “88” are used for phage display on p8 coat protein [26,86,87,88].

#### 3.1.2. T4 Phage Display Platform

The display of fusion peptides/proteins on T4 phage has emerged as a promising tool to overcome the limitations of phage display platforms employing filamentous phages. For instance, one of the drawbacks of filamentous phage display is the small size of the peptides displayed on the major coat protein (6–10 amino acid residues). Larger polypeptides can only be displayed on minor coat proteins but at very low copy numbers. Moreover, during phage amplification, synthesized coat proteins are inserted into the inner cell membrane where virion assembly and export occur. Due to the membrane-mediated nature of this process, fusion proteins that cannot cross the cell membrane will not be displayed on the phage surface. The secretion system of *E. coli* may also prevent the display of some peptides that are toxic to bacteria and may also create the problem of achieving correct folding of the displayed protein [91,92,93,94]. However, T4 phage uses a lytic life cycle for reproduction in which phage assembly takes place inside the infected cell and, afterwards, progeny phages are released by cell lysis. This feature of T4 enables the display of a broader range of proteins with different size, structure, and biological functions that may not be possible with filamentous phage display [32,95].

In T4 phage display, Soc and Hoc are used for the fusion of foreign proteins (Figure 2C). Because both Soc and Hoc sites can be used simultaneously if desired, it has been shown that higher copy numbers of fusions on the phage surface can be achieved by display on both sites (Soc and Hoc) [96,97]. Foreign proteins can be displayed on T4 phage by in vivo and in vitro approaches. The in vivo approach can be performed in different ways, one of which is based on the integration of a modified *soc* gene into a *soc*-deleted T4 genome through a modified positive selection plasmid. In this type of plasmid, the *soc* gene is flanked on its 5′ side by a 3′ portion of the T4 lysozyme gene (e’), and on its 3′ side by a 5′ part of another T4 gene (denV’), which allows homologous recombination between the phage and the plasmid. Integration of the *soc* fusion gene into the T4 genome allows the expression and in vivo binding of fusion proteins to the phage capsid [93]. Alternatively, Soc and Hoc fusion proteins can be incorporated to the phage capsid through a natural assembly process in host bacteria expressing the fusion proteins from a designed expression vector. Upon infection of bacteria with T4 phage strains having defective *soc* or *hoc* genes, fusion proteins are expressed and assembled onto the phage capsid [98,99].

Although in vivo approaches have been widely used to display different proteins on the surface of T4 phage, they are limited to the display of single components, such as a peptide, a domain, or a protein. Limitations in displaying multiple components and large domains arise from the fact that the expression and assembly of the foreign proteins occur during phage infection. This lytic phage cycle leads to problems such as the loss of critical epitopes due to nonspecific proteolysis, low and variable copy number of displayed proteins due to the variations in intracellular expression, structural heterogeneity due to aggregation of the expressed proteins, insolubility, and improper folding [93,100]. Thus an in vitro approach has been developed to overcome these drawbacks for efficient and controlled display of large proteins on the phage surface. In this approach, foreign proteins fused to Soc and Hoc proteins are overexpressed in bacteria and purified. The high affinity interactions between Hoc/Soc proteins and the phage capsid enable in vitro assembly of purified proteins on Hoc- and Soc-defective phage, which is performed by simply mixing the components. Therefore, the in vitro approach results in a phenotype no longer connected to the genotype of the engineered phage, which contrasts to the in vivo approach. An attractive feature of the in vitro approach is that the expression of Hoc/Soc fusion proteins is not restricted to *E.coli* or another specific host, thus any expression system can be used for production of fusions. Consequently, functionally well-characterized and conformationally homogenous fusion proteins are produced and displayed on phage capsids. Additionally, the copy number of displayed proteins can be controlled by changing the ratio of protein to capsid binding sites. It is worthy to note that the in vitro approach also allows customized engineering of T4 phage to display multiple proteins on the same capsid [101,102,103].

#### 3.1.3. T7 Phage Display Platform

The T7 phage capsid is composed of gp10A and gp10B, which have been employed to display peptide moieties (Figure 2B). While high display numbers can be achieved for peptide sequences shorter than 50 amino acids, only a few copies of larger proteins (<1,200 amino acids) are displayed per capsid [95]. Therefore, the T7 phage display platform becomes favorable for the display of large proteins. As for T4, T7 phage display vectors also overcome certain limitations of filamentous phage display platforms. The lytic nature of T7 phage eliminates the need for protein export and enables the display of a broad diversity of proteins on the phage’s surface [104,105]. Moreover, the lytic life cycle shortens the time required for biopanning steps and thus accelerates the selection from the phage library [106]. Unlike many other phage display systems, the coat proteins of T7 phage are anchored to the phage through their N-termini, which makes their C-termini available to display the peptide moieties. This feature makes T7 phage attractive to develop recognition moieties for targeting protein domains that preferentially interact at their N-termini [107].

#### 3.1.4. λ (lambda) Phage Display Platform

Lambda phage libraries are used as another lytic phage display platform to overcome the limitations of filamentous phage display systems, and employ either the gpV tail protein or the gpD decorative capsid protein (Figure 2D). The tail tube consists of 32 disks, each containing six subunits of gpV protein. The small C-terminal domain of gpV protein is exposed and allows the expression of protein moieties as fusions, however, the fusion proteins are only displayed at low levels (ca. one molecule per phage particle) [108]. On the other hand, the gpD protein enables the display of fusions on both its N- and C-termini [109]. While the level of display depends on the size of the fusion, large tetrameric proteins can be displayed at lower levels compared to the small protein domains [110]. In order to overcome low display levels of lambda phage, two different approaches have been investigated. The first approach uses a two-gene system, where both wild type gpD protein and gpD fusion coat proteins are co-packaged into the lambda’s head and generate mosaic phage particles expressing both proteins [111,112]. In the second approach, nonsense suppression is used to control the level of the fusion protein. A stop codon is introduced between the gene of gpD protein and its fusion partner. As the gene of gpD protein cannot be fully suppressed, some wild type protein will be also expressed and displayed on phage surface [110,113].

#### 3.1.5. MS2 Phage Display Platform

The major coat protein of MS2 can be used to display foreign proteins/peptides on its surface with high copy number. While the insertion of peptide sequences can be accomplished at different regions of the coat protein, the short hairpin loop between two β-strands (βA and βB) of the protein subunits has been the most commonly used part, as it allows the display of the peptide sequences on the outer surface of the phage (Figure 2E) [114,115,116,117,118,119]. Small peptide sequences can be displayed as N-terminal extensions of the coat protein subunits, which protrude from the phage surface as well. However, some deletions and base-substitutions can be observed due to the lack of genetic stability. The genetic stability of the insert highly depends on the structure of the RNA hairpin loop encoding the insert and it is determined by the choice of the nucleic acid sequence [120].

### 3.2. Phage Display-Derived Therapeutics

Phage display is one of today’s most important drug discovery technologies. It allows the identification of a broad range of potential therapeutic peptides and antibodies, as well as polypeptides with a variety of functions (protease inhibitors [121,122], minimizing proteins [123], novel scaffolds [124], and DNA binding proteins [125]). Amongst all of these, monoclonal antibodies (mAb) have received considerable attention. The generation of mAbs started with the discovery of the hybridoma technology by Köhler and Milstein in 1975, in which hybrid cells were developed by the fusion of B-cells from immunized animals with myeloma cells to produce antibodies [126]. 10 years later, the first approved mAb, muromonab- CD (Orthoclone OKT3^®^) [127], was produced using this technology. However, due to the non-human origin of this mAb, a significant percentage of patients developed immune responses, which called into question the safety and efficacy of the non-human mAb therapy [128]. In the late 1980s, recombinant DNA technologies allowed the humanization of non-human mAbs to make them more similar to antibodies within the human body [128]. Starting in the 1990s, human antibodies were produced by in vivo immunization and hybridoma technology using transgenic mice or rats containing the human antibody gene repertoire, or parts of it [129,130,131]. However, the immunization of transgenic animals could not be used for the production of in vivo antibodies for all types of antigens (e.g., unstable, conserved, and toxic antigens). These limitations impose the use of other alternatives such as in vitro selection technologies, which can be used to discover antibodies towards almost every type of antigen, as they do not depend on immunization.

As such, in vitro display technologies such as phage display, yeast display, ribosome display, bacterial display, mammalian cell-surface display, mRNA display, and DNA display have been used for antibody discovery [132]. Among these, phage display is the most widely used for antibody selection [133]. Its use has resulted in the discovery of over 80 mAbs that have entered clinical trials [134]. In 2002, adalimumab became the first phage display-derived mAb to have been granted market approval, and was also the first approved human mAb [131]. Small recombinant antibody fragments (e.g., scFv) [135,136] or fragment antigen-binding (Fab) [137,138] are also commonly selected by phage display, in addition to full antibodies [139]. A selection of FDA-approved phage display-derived antibody therapeutics are summarized in Table 2. Four exceptions in this table are the non-antibody peptides ecallantide (Kalbitor^®^), Romiplostim (Nplate^®^), albigutide (Tanzeum^®^), and Xyntha purification peptide. Antibody libraries are huge collections (>10^10^) of antibody genes encoding antibodies with unknown properties. These are an essential resource for antibody discovery by phage display and other in vitro selection technologies [133]. Depending on their source of origin, antibody libraries are classified as immune libraries and universal libraries. Immune libraries containing affinity-matured antibodies [133] are constructed using donors (humans or animals) who have received immunization, have been infected or a chronically-diseased, or those suffering from cancer [140]. Affinity maturation is achieved by mutation and clonal selection in which mutated antibodies with higher antigen-binding affinity are enriched [141]. However, extensive or hypermutation may increase the risk of immunogenicity [133]. Of course, it is not possible to construct immune libraries for each disease due to ethical issues, high cost, and laborious procedures. One solution to this problem is to use universal libraries that are generated by a source for which the immune system had not been activated to recognize a specific antigen (naïve) [132]. Moreover, due to the lack of affinity maturation, these universal libraries have low risk of immunogenicity [133]. In principle, a universal library can be applicable to mAb selection of any type of antigen. This is because the library comprises a high variability of antibody genes and comprises antibody genes from many donors [132]. Universal libraries are further sub-classified as naïve, semi-synthetic, or fully-synthetic. Naïve antibody libraries are constructed from the natural human IgM repertoire (i.e., from not intentionally immunized donors) [132,133]. Examples of naïve universal libraries are the human Fab library constructed by de Haard and colleagues at Dyax (now Shire) [137], the scFv libraries from Cambridge Antibody Technology (CAT) [142,143], scFv and Fab libraries from XOMA59, and the HAL scFv libraries [135,144]. Fully synthetic libraries are constructed to include synthetic genes derived from known (human) antibody frameworks with the capacity to generate a large diversity in appropriate regions [145]. Semi-synthetic libraries are a combination of natural (i.e., donor-derived antibody) and synthetic antibody sequences [138]. A combination of naïve and synthetic repertoires was used for the Dyax FAB310 library. Fully-synthetic libraries were developed by MorphoSys [133]. In addition, a particular type of antibody library is generated during guided phage display selection of human antibody using a non-human original antibody sequence. This strategy has been used for humanization and the discovery of fully human antibodies with similar properties to the murine antibody template, such as adalimumab [146]. 

Overall, the identification of mAbs and mAb derivatives by phage display technology was a breakthrough that has enabled the isolation of human antibodies towards many types of antigen without immunization. Since then, it is one of the main platforms for generation of human therapeutic antibodies together with transgenic immunized mice, antibody humanization techniques, and single B-cell expression cloning [169].

### 3.3. Phage Display Selection of Peptide Binders for Biomineralization/Self-Assembly of Inorganic Materials

Reports showing that peptides sequences displayed on the outer surface of *E.coli* could recognize and specifically-bind to metal/metal oxide surfaces (e.g., gold, iron oxide, and chromium) inspired research to extend this concept to phage display [170,171]. The first application of phage display libraries to evolve peptide sequences binding to inorganic substrates was performed for a range of semiconductor surfaces with the motivation of directing nanoparticles to specific locations on semiconductor structures for the fabrication of complex, sophisticated electronic materials [172]. This achievement led to research in phage display selection of material-binding peptides. Several peptide sequences with affinity to different materials (e.g., platinum, palladium, titanium, silicon, silver, gold, zinc sulfide, cadmium sulfide, graphite, calcite, indium phosphide, chlorine-doped polypyrrole (PPyCl), and carbon nanotubes) have been identified [78,173,174,175,176,177,178,179,180,181,182,183,184,185,186,187].

Peptides selected by phage display have not only been used to achieve binding, but have also been employed to direct the mineralization of nanomaterials. This approach is inspired by the process of biomineralization of materials in Nature by living organisms. Several biominerals are formed in a biologically-controlled manner under mild conditions and include calcium phosphate minerals in teeth and bone, silica in sponges, and magnetic particles of magnetite (Fe_3_O_4_) or greigite (Fe_3_S_4_). Recent interest in biomineralization has grown as it offers a greener and cheaper alternative to inorganic synthesis of materials, which usually requires high temperatures and harsh chemical reagents [188]. Peptide sequences that can facilitate biomineralization have been identified by phage display selection against target materials, considering that some selected peptides with binding affinity to the target material can nucleate or promote the formation of these materials. M13 phage was used to select several peptide sequences capable of recognition and nucleation of different materials like zinc sulfide (ZnS), cadmium sulfide (CdS) nanocrystals, iridium oxide (IrO_2_), cobalt platinum (CoPt), and iron platinum (FePt) materials [189,190,191,192,193]. Although the specific interactions between the peptide sequences and the ions are crucial for the nucleation and growth of these materials, the uniform conformation of the displayed peptides on phage surface is also mentioned to be important for controlled crystallization of the materials with single-crystal nature. While not directly used as therapeutics per se, the ability of phage display technology to identify peptides that bind with affinity to inorganic substrates has led to the use of viruses as building blocks of functional structures for drug delivery applications. In the following section, the techniques used for fabrication of virus-based drug carriers and the approaches to design virus-based drug delivery systems are presented.

## 4. Virus-Based Drug Delivery Systems

### 4.1. Encapsulation/Decoration with External Cargos

One of the most attractive features of plant viruses is that the coat proteins can self-assemble around synthetic materials, offering a stable and biodegradable delivery platform for various compounds. The self-assembly of virus coat proteins around cargo molecules can be achieved in different manners, depending on the virus. The interaction of coat proteins with a specific sequence of viral RNA (RCNMV) or a negatively charged material (BMV, CCMV, HCRSV) to replace the negatively charged RNA is crucial for some viral platforms to encapsulate foreign materials [61,194,195,196]. However, some virus particles can form empty capsids in the absence of any genetic/external material (CPMV) by simple co-expression of essential viral proteins in plant cells [197]. Moreover, the pores present on the viral capsids can be employed to encapsulate small molecules [60]. However, the size of capsid pores may not be sufficiently large for diffusion of small molecules in all virus platforms. In this case, pore formation can be induced by depletion of capsid-integrated divalent ions for some viral particles (e.g., RCNMV) [198]. Like many plant viruses, the relatively large interior volume of MS2 phage capsid provides an interesting platform to load molecules or materials. Spontaneous assembly of MS2 coat proteins in the presence of RNA sequences enables the loading of RNA-conjugated functional materials inside the phage capsid. Encapsulation of phage RNA is mediated by a 19-nucleotide RNA stem-loop, the so-called pac site, which specifically interacts with coat proteins. Inspired by this mechanism, molecules of interest, such as antisense oligodeoxynucleotides, antisense RNAs, quantum dots, drugs, and toxins, have been conjugated with a pac site to initiate the assembly of coat proteins and to achieve packaging within the capsid [199,200,201]. The reassembly of coat protein dimers around functional moieties is another strategy employed to encapsulate external cargos within the MS2 capsid. In this approach, phage capsids are first disassembled into dimers with acetic acid. Then, reassembly is initiated with negatively charged DNA/polymer/amino acid tags conjugated to a cargo molecule, due to their electrostatic interactions with the interior positively-charged capsid dimers. While the presence of highly-negatively charged cargo (ca. DNA-coated gold nanoparticles) is sufficient to initiate the reassembly, it might be necessary to add protein stabilizing osmolytes to increase the yield of reassembly around protein cargos, such as enzymes [202,203].

The ability to empty the T7 phage capsid of its genetic material opens up the opportunity to use these hollow phage heads as cages for encapsulation of foreign materials. It has been demonstrated that the phage DNA can be released from the capsid by applying osmotic pressure, and the empty capsids then filled with precursor ions for mineralization [204]. The self-assembly of nanoparticles on the capsid of T7 has also been reported in the literature. This was accomplished by introducing functional moieties (biotinylation peptide, gold binding peptide) onto the coat protein through genetic engineering, and were used to assemble quantum dots and gold nanoparticles [205,206]. M13 and MS2 phage capsids have also been used as templates to assemble a variety of materials (e.g., ^64^Cu, Gd^3+^, drugs, single wall carbon nanotubes, iron oxide nanoparticles and fluorophores) with high copy number by means of genetic/chemical modification of the coat proteins for site-specific material conjugation [84,207,208,209,210,211].

The capsid of T4 phage has also used as a bio-scaffold to fabricate functional materials. The fabrication of such nanostructures typically relies on the reduction of metal nanoparticles on the capsid surface, and is generally performed in two steps. T4 phage are initially incubated in a solution of metal salts and then the metal ions that interact with phage coat proteins are reduced by dimethylaminoborane [212]. Although this approach has been applied to synthesize different phage-templated metal nanostructures (e.g., gold, platinum, rhodium, cobalt, iron, palladium, and nickel), the mechanism of metal nanoparticle formation on phage coat protein remains unknown [213]. It has been suggested that the side-chains of the surface-exposed amino acids on coat proteins interact with the metal ions and mediate the nucleation and organization of the nanoparticles on T4 capsid [214].

### 4.2. Drug Delivery Systems

The extensive use of phage in drug discovery and for the identification of binding partners to various targets have also led researchers to consider using the virus itself as a targeting probe/nanocarrier in medicine. Genetically/chemically-modified viruses that display targeting peptides or synthetic functional molecules have been used as building blocks to design self-assembled nanostructures for drug delivery and the treatment of diseases. In particular, the reported ability of filamentous phages to penetrate to the central nervous system, which is difficult for most of the drug molecules and drug delivery systems due to the relative impermeability of blood-brain barrier, may contribute to making viruses promising drug delivery platforms [215].

#### 4.2.1. Anti-Cancer Drugs

**Figure 3:**
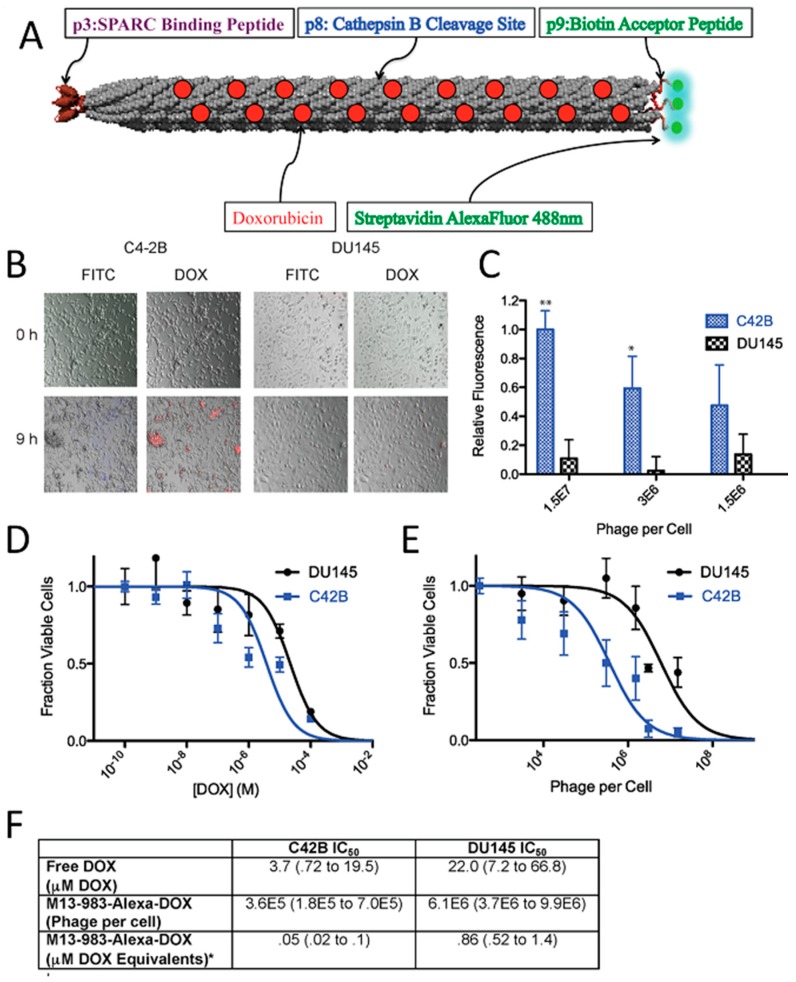
(**A**) Schematic of M13-983 Phage. The red dots along the phage coat represent doxorubicin (DOX) attached to p8. p3 displays a peptide with affinity for SPARC (Secreted Protein, Acidic and Rich in Cysteine), and p9 can be enzymatically biotinylated and loaded with streptavidin-functionalized fluorophores AlexaFluor 488 nm. (**B**) Overlay of brightfield and fluorescent images of SPARC positive C42B cells (first and second column) and less expressing SPARC DU145 cells (third and fourth column) incubated with M13-983-Alexa-DOX at 0 h (top row) and 9 h post-treatment (bottom row). FITC channel represents fluorescence from Alexa Fluor 488, and DOX is designated by red fluorescence from DOX uptake. C42B samples showed increased fluorescence of phage uptake, indicated by green fluorescence (bottom row, first column) and DOX uptake (bottom row, second column) as compared to DU145 cells after 9 h. (**C**) Targeted uptake measured by quantifying fluorescence intensity (** *p* < 0.001; * *p* < 0.01). C42B consistently shows higher fluorescence intensity than DU145, confirming the observations in panel B. Higher phage concentrations report larger differences between C42B and DU145 fluorescence. (**D**) Cell viability of C42B and DU145 as a function of free DOX. (**E**) Cell viability of C42B and DU145 cell lines as a function of increasing M13-983-Alexa-DOX. All samples were run in triplicate and error bars represent standard deviations. (**F**) IC50 values for C42B and DU145 are given with the 95% confidence interval given in parenthesis. *Based on 257 DOX particles per phage (Adapted with permission from [84], Copyright American Chemical Society, 2012).

The most common virus-based drug delivery platforms are based on chemotherapeutics, and the viral particles are employed as carriers for small drug molecules. In this manner, doxorubicin, which is a clinically approved anticancer agent, has been extensively studied. Viral particles have been employed to improve the drug efficacy and reduce systemic toxicity. In order to create a linker with high serum stability and sensitivity to enzymatic hydrolysis by cysteine protease (cathepsin-B; present within the lysosomes of target cells), cathepsin-B cleavable DFK peptides were displayed on the p8 coat proteins of filamentous phage and employed to attach doxorubicin with a high copy number (~3500) via carbodiimide coupling chemistry. Although, direct conjugation of the drug to the coat proteins yielded higher numbers of drug molecules per phage (~10,000), the engineered drug-release mechanism significantly improved the potency of the carrier as a result of release of the drug at the targeted cells [216]. Considering that all five coat proteins of M13 can be used for phage display, it is possible to introduce more than one functionality onto the surface of the phage to achieve new properties. By using three different coat proteins of M13, a phage-based therapeutic platform was designed for simultaneous prostate cancer imaging and targeted drug delivery [84]. In this system, doxorubicin was conjugated to the major coat protein while other coat proteins (p3 and p9) were used to display cancer-targeting peptides and fluorophores (imaging agent), respectively (Figure 3). The phage-based therapeutic enabled cancer cell targeting, imaging, and drug delivery.

As an alternative approach, rather than loading drug molecules onto viral particles, drug-loaded materials can be modified with viruses for targeting purposes. For instance, M13 phage modified with folic acid to target cancer cells, was attached onto a biodegradable polymer (poly(caprolactone-b-2-vinylpyridine, PCL–P2VP) particles that were loaded with doxorubicin [217]. By providing a large surface area with control over the spacing and orientation, phage particles enabled multivalent target-receptor interaction and improved targeting. Indeed, in vitro studies with human nasopharyngeal cells showed that doxorubicin-loaded phage coated polymer particles had significantly higher cellular uptake and selectivity in comparison to free drug.

Encapsulation of doxorubicin within the cavity at the center of MS2 has been an alternative strategy to improve the delivery of the drugs to target cells. It has been demonstrated that reduced intracellular accumulation of doxorubicin in human hepatocellular carcinoma cells (Hep3B) due to the moderate P-glycoprotein levels can be overcome by loading empty MS2 virus capsids with drug molecules [199]. As targeted drug-loaded viral particles are internalized via receptor-mediated endocytosis, they can circumvent efflux mechanisms of P-glycoproteins and can kill cancer cells at lower drug concentrations (20-fold improvement) compared to free drugs. Moreover, encapsulation of doxorubicin inside the MS2 viral capsid demonstrated significantly different time-dependent cytotoxicity. Indeed, while the free drug was highly toxic to all studied cell types exposed to the drug for 24 h, encapsulation of the drugs within the targeted viral particles showed a high degree of specificity towards Hep3B cells, with an >80% reduction in cell viability. In contrast, the viability of non-targeted cells after a 7 days exposure to the encapsulated drug remained relatively unaffected. Similar results were observed for doxorubicin-loaded cancer targeted RCNMV, CMV, and HCRSV particles, which reduced the cytotoxicity of the drug in non-targeted cells, due to the specific cell uptake in target cells [60,62,218]. CMV drug carriers exhibited a sustained in vitro drug release profile over 5 days. The efficacy of doxorubicin-loaded viral particles in tumor-bearing animal models have been studied, as well. Regarding doxorubicin-loaded TMV particles, treatment showed significant delay in tumor growth and increased survival due to the efficient tumor accumulation of the drug carrier platform while free doxorubicin had no effect on tumor burden or survival [219]. PEGylated doxorubicin-loaded PVX particles also showed similar efficacy in animal models. Tumor growth rates were significantly lower compared to free doxorubicin [220]. Cardiotoxicity of doxorubicin was also studied in viral drug delivery systems in order to demonstrate the efficiency of targeted drug delivery along with the prevented drug release within the heart. Johnson grass chlorotic stripe mosaic virus (JgCSMV, a member of the family *Tombusviridae*), another plant virus recently gained attention as an alternative viral platform, was loaded with doxorubicin and drug delivery efficiency of doxorubicin loaded JgCSMV has been investigated in MCF-7 tumor-bearing athymic mouse models [221,222]. The study showed that tumor volume of the mice treated with doxorubicin loaded JgCSMV was 2.22 times smaller than the control group which was not treated with any drug. More interestingly, hearts of the mice treated with doxorubicin loaded JgCSMV and untreated negative control mice showed no significant pathological changes while thrombi were observed in hearts of the mice treated with free doxorubicin. In addition to rod-like filamentous structures, TMV coat proteins can be self-assembled into stable disk-shaped particles, which expands the shape library of the protein-based nanomaterials for drug delivery. Delivery of doxorubicin molecules to glioblastoma cells in vitro demonstrated that TMV disks could provide a promising nanocarrier platform resulting in significant cell death after 72 h of incubation while the cells incubated with TMV disks alone showed ~100% viability [64].

**Figure 4:**
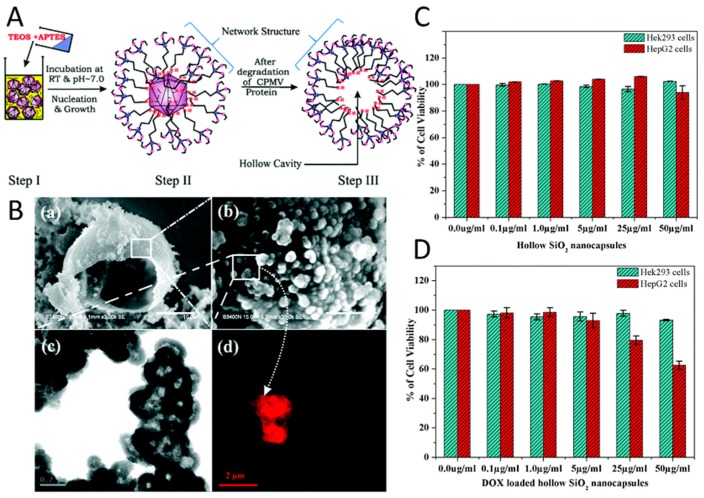
(**A**) Schematic representing the proposed synthesis mechanism of the CPMV-templated mesoporous silica nanocapsules in three steps (Step I, Step II and Step III). (**B**) (a) SEM image of single hollow capsules formed through the self-assembly of hollow SiO_2_ nanocapsules synthesized in the presence of CPMV, (b) surface textures of the same formed by the self-assembly of nanoparticles. (Scale bar 10.0 μm and 1.0 μm, respectively), (c) TEM of hollow SiO_2_ nanocapsules (shown in (b), scale bar 0.2 μm), and (d) confocal microscopy image of hollow SiO_2_ nanocapsules loaded with Rh6G (a small fluorescent molecule) (scale bar 2 μm). Cytotoxicity assay of Hek293 (Human embryonic kidney cell line) and HepG2 cells (Human carcinoma cell line) (**C**) with mesoporous SiO_2_ nanocapsules free from drugs and (**D**) nanoformulated hollow SiO_2_ nanocapsules (doxorubicin (DOX)-loaded hollow SiO_2_ nanocapsules) of different doses (Adapted with permission from [223], Copyright The Royal Society of Chemistry, 2015).

The formation of hollow mesoporous silica nanocapsules around CPMV viral templates has been another approach to control drug delivery from a viral scaffold. APTES ((3-aminopropyl) triethoxysilane) and TEOS (tetraethyl-orthosilicate) were used to form a silicate network around viral capsids due to electrostatic interactions between the carboxyl/carbonyl groups of CPMV and amine groups of silicates (Figure 4A) [223]. In order to create a hollow cavity, the capsid proteins of viruses were denatured by increasing the temperature to 40 ºC for a period of 24 h, which enabled their diffusion out from the core. The pores within the silica nanocapsules enabled the loading of doxorubicin into its cavity and their release by diffusion (Figure 4B). The fabrication of surfactant free, hollow mesoporous silica nanocapsules provided high drug stability as a result of slow decomposition of the drug molecules, which were protected inside the capsules and their regulated sustained release from the pores. The in vitro efficacy of the virus-templated mesoporous silica nanocapsules were investigated by utilizing Hek293 and HepG2 cell lines, where the raise in drug dosage resulted in an increase in the cell survival (Figure 4C–D) and showed the efficiency of the platform.

In addition to doxorubicin, other anticancer pro-drug/drug molecules have also been successfully loaded inside the viral nanoparticles and their drug delivery efficiencies in cancer cells have been evaluated. Proflavine, mostly known as a bacteriostatic, is one such compound that has shown antiproliferative activity in cancer cells and tumors due to its intercalation into DNA [224]. Loading of proflavin within the CPMV particles has been achieved through the diffusion of drug molecules into the viral capsids. The loading mechanism of the drug within the capsid was explained by the genetic material inside the capsid acting as a sponge that absorbs the drug molecules. Drug delivery studies have shown that the interaction of proflavine with viral RNA was reversible and enabled the release of the drug molecules in several cancer cell lines (HeLa (cervical cancer cells), HT-29 (colon cancer cells), and PC-3 (prostate cancer cells)) while no cargo release was observed in cell-free medium.

Cisplatin (cis-[Pt(NH_3_)_2_Cl_2_]) is another anticancer drug that has been efficiently delivered to cancer cells via viral particles. TMV particles have been decorated with mannose and lactose moieties to specifically target the galectin-rich human breast cancer cell line MCF-7 and asialoglycoprotein receptor (ASGPR) over-expressing hepatocellular carcinoma cell line HepG2, respectively. For this purpose, alkyne modified TMV particles were modified with azido sugar derivatives via the copper(I)-catalyzed azide–alkyne cycloaddition. The interior capsid surface of the TMV particles was covalently modified with cisplatin molecules through a stable chelate structure with the carboxyl groups of glutamate residues, and the drug later slowly released and resulted in enhanced apoptosis efficiency in specific targeted cell lines [225]. A similar drug delivery approach has also been employed for the treatment of ovarian cancer cells with platinum resistance, which may appear at the onset of disease or develop in response to platinum-based chemotherapy. As cisplatin offers greater efficacy than its analogue carboplatin, it is crucial to develop alternative drug delivery platforms employing cisplatin with reduced toxicity. It has been reported that the delivery of drug molecules conjugated to the interior surface of TMV provided a platform capable of circumventing the resistance mechanisms in platinum resistant ovarian cancer cells and restoring efficacy of cisplatin treatment at low concentrations. Loading of TMV particles with cisplatin was achieved through electrostatic interactions between the deprotonated interior glutamic acid residues of the capsid proteins with positively-charged cisplatin molecules, which were produced via a reaction with silver nitrate. The enhanced efficacy of cisplatin-loaded TMV particles in ovarian cancer cells suggests that encapsulation of cisplatin in viral particles increased the rate of drug uptake/retention as well as DNA damage inside the cells [226].

#### 4.2.2. Protein Therapeutics

The plant hormone indole-3-acetic acid (IAA) is a prodrug used in a virus-based drug carrier to treat human prostate cancer cells. IAA generates a radical upon reaction with horseradish peroxidase and produces radical-dependent cytotoxicity as well as cell death. The viral drug carrier was designed by engineering M13 phage to display a short peptide to enhance prostate cancer cell recognition/penetration. Moreover, a NeutrAvidin–horseradish peroxidase conjugate was attached to the p9 phage coat protein of M13 fused with a biotinylated peptide [227]. The treatment of cancer cells with this system led to a significant reduction in cell viability due to intracellular delivery. This virus-based prodrug activation approach has also been investigated for tamoxifen, which is one of the most widely used prodrug in the treatment of hormone-dependent breast cancer [228,229]. Tamoxifen is mainly metabolised in the liver by cytochrome P450 (CYPs) enzymes, resulting in the active drug. Encapsulation of CYPs inside cancer-targeting CCMV particles was suggested as a pro-drug activation strategy to increase the drug efficiency as well as to reduce the severe side-effects of the drug in normal cells. In order to encapsulate the CYPs, viral particles were disassembled and then reassembled in the presence of the enzyme molecules. The electrostatic interactions between the negatively charged CYPs and the positively charged interior of the viral capsid was used as a driving force to internalize the enzyme molecules within the viruses. Preliminary studies have shown that CYPs encapsulated within the viral particles maintained their activity, though the catalytic activity was one order of magnitude lower compared to the activity of the free CYPs. The decrease in enzyme activity was attributed to deleterious effects of crowding inside the capsid cavity, diffusion of the substrate into the virus capsid, and improper orientation of the active site of the enzyme.

#### 4.2.3. Antibiotics

Antibiotics are another group of drugs that have been loaded in viral-based drug carriers. It has been reported that the conjugation of many copies of the drug molecule onto the phage’s major coat proteins increased potency by creating a microenvironment around bacterial cell with a locally high drug concentration. Treatment of gram positive pathogenic bacteria *Staphylococcus aureus* with the low potency antibiotic chloramphenicol, well-known for its toxicity to blood cells, conjugated to the fd phage retarded the growth of bacterial cells ~20-fold more efficiently than free chloramphenicol [230].

#### 4.2.4. Photodynamic and Photothermal Therapy

Efficient delivery of drug molecules to targeted cells is also important for photodynamic therapy (PDT) and photothermal therapy (PTT). PDT relies on the activation of a photosensitizer by light in the presence of oxygen, which produces reactive oxygen species (ROS). As cell damage occurs due to the reaction of ROS with cellular components, it is important to deliver the photosensitizers to the targeted cells and avoid their nonspecific delivery to the healthy cells. The nonspecific dispersal of the photosensitizer throughout the body creates sensitivity in patients. For instance, sunlight must be avoided for several weeks following treatment. Moreover, the insolubility of many photosensitizers in physiological solutions is another problem encountered in PDT-related drug delivery systems and it is necessary to develop new platforms to address this challenge. For this purpose, icosahedral CCMV particles have been explored for PDT by dual labelling with both cell-targeting moieties and photosensitizers. The surface coat protein of the virus was genetically-modified to display a cysteine residue, used to attach the photosensitizer Ru(bpy_2_)-5-iodoacetoamino-1,10-phenathroline(phen-IA). An antibody specific for *Staphylococcus aureus,* was chemically conjugated to the lysine residues of the coat proteins [57]. PDT studies using this system showed that the photosensitizer-labelled CCMV particles were more efficient in killing *S. aureus* cells compared to free photosensitizer due to the enhanced cell targeting ability. On the other hand, the cell killing efficiency of the photosensitizer-labelled CCMV particles was approximately the same as that achieved using and anti-*S.aureus* antibody-photosensitizer conjugate. While the number of photosensitizers per binding event for the CCMV platform (~70) was significantly higher than the antibody-photosensitizer conjugate (~2), it was suggested that the large size of the CCMV particles significantly reduced the proximity of photosensitizer to the cell surface, which is an important factor in efficacy due to the very short diffusion length of singlet oxygen. The encapsulation of photosensitizers within the capsid cavity of CCMV particles has been another approach for PDT. Water-soluble zinc phthalocyanine (ZnPc) was encapsulated inside CCMV by two different routes: i) self-assembly of coat proteins dimers around aggregated ZnPc molecules at neutral pH; and ii) diffusion of ZnPc molecules into empty CCMV particles through capsid pores at acidic pH [231]. Although the potential use of ZnPc-loaded CCMV particles as PDT delivery system was tested on macrophages and resulted in cell death, further studies are required to evaluate the efficiency of this platform as targeted PDT delivery system for cancer therapy. Zn-EpPor (5-(4-ethynylphenyl)-10,15,20-tris(4-methylpyridin-4-ium-1-yl)porphyrin-zinc(II) triiodide) is another photosensitizer used in viral PDT systems. The interior channel of TMV particles were loaded with the photosensitizer by exploiting electrostatic interactions between the negatively-charged amino acid residues of virus and the positive charge of Zn-EpPor [232]. Zn-EpPor-loaded TMV particles were stable and possessed a good shelf-life (drug release was not observed during one-month storage at 4°C in 0.01 M potassium phosphate buffer, pH 7.0). Cellular uptake and drug efficacy studies were performed with melanoma cancer cells. The release of the photosensitizer was suggested to occur inside the acidic endolysosomal compartment, which caused protonation of TMV’s interior carboxylic acid groups resulting in drug release. Zn-EpPor-loaded TMV particles showed enhanced cell killing efficacy compared to free Zn-EpPor molecules, which was attributed to the increased cellular uptake of photosensitizer as a result of their delivery within the TMV particles. Recently, the effect of photosensitizer’s charge on drug loading efficiency of TMV particles was investigated in a study where different zinc porphyrin (Zn-Por) formulations (monocationic (Zn-Por^1+^), dicationic (Zn-Por^2+^), tricationic (Zn-Por^3+^), and tetracationic (Zn-Por^4+^)) were employed [233]. While the tricationic formulation demonstrated the highest loading efficiency (~600 molecules/TMV), the results were attributed to the combined effect of electrostatic and hydrophobic/hydrophilic interactions; higher positive charge was suggested to result in better stabilization of the photosensitizers inside TMV particles due to the electrostatic interactions with the deprotonated carboxylate residue of glutamic acid at pH 7.8. Moreover, the increased hydrophobic nature of the monocationic and dicationic Zn-Por formulations with their electrostatic properties resulted in aggregation and reduced the loading efficiency. In order to develop a targeted drug delivery system, F3 peptide was conjugated to the surface proteins of TMV particles to target a shuttle protein, nucleolin, overexpressed on HeLa cells. Zn-Por^+3^ loaded TMV-F3 particles accumulated on the cell membrane and showed a fivefold increase in cell killing efficacy compared to the free drug. The results are explained with some possible mechanisms which are the cell toxicity through cell membrane disruption by light activation, the release of the photosensitizers at the cell surface and favored cell uptake of the Zn-Por molecules due to their positive charge. The self-assembly of drug-loaded liposomes on M13 phage has been another PDT delivery approach using phage as a nanocarrier [234]. Cationic liposomes were loaded with zinc phthalocyanine (ZnPc) and were assembled on M13 phage displaying eight glutamic acid residues on the p8 major coat protein, via electrostatic interactions. Phage-templated drug-loaded liposomes had enhanced excited singlet oxygen generation efficiency and were able to internalize in breast cancer cells. These two properties make phage-liposome complexes a promising tool for targeted drug delivery given that this property can be introduced by displaying targeting peptides on the minor coat proteins of phage. Moreover, the phage template can stabilize the liposomes in biological media against flocculation and can help the delivery of the content loaded inside the liposome to specific targets.

**Figure 5:**
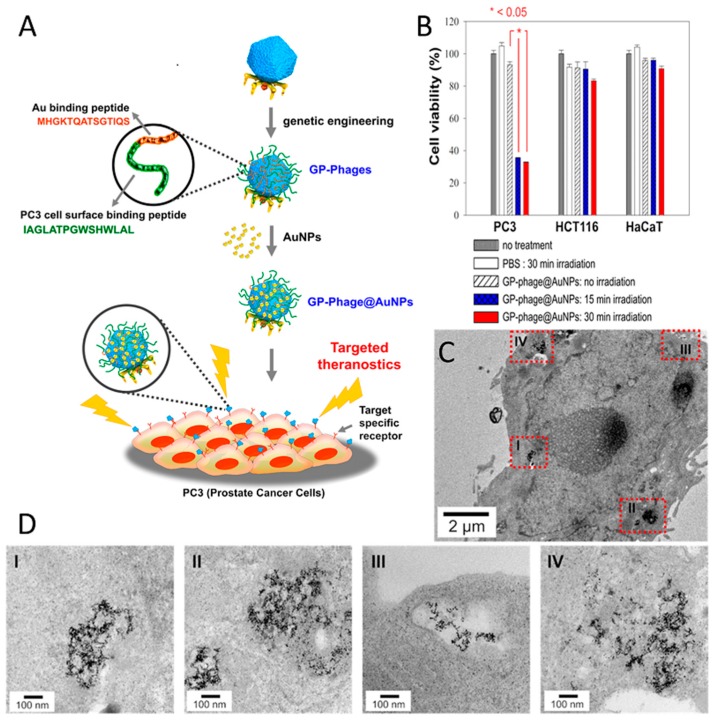
(**A**) Schematic illustration of cancer-selective photothermal therapy via prostate cancer-targeted intracellular delivery of T7-templated AuNP nanoclusters, where T7 phages are genetically modified to display gold-binding and prostate cancer cell-targeting peptides on the viral surface. (**B**) The viability of each cell line (prostate cancer cell (PC3), human colorectal carcinoma cells (HCT116), and normal cells (HaCat)) by photothermal effects of T7-templated AuNP nanoclusters. (**C**,**D**) TEM images of T7-templated AuNP nanoclusters internalized within PC3 cells in ultrathin section specimens. The cells were treated with T7-templated AuNP nanoclusters for 5 h, followed by medium replacement and additional incubation for 20 h (Adapted with permission from [206], Copyright American Chemical Society, 2015).

Photothermal therapy (PTT), so-called hyperthermal therapy, employs gold nanoparticles as a heat source for inducing cell damage as a result of a light-to-heat conversion process. As gold nanoparticles generate local heating under light illumination, efficient delivery of gold nanoparticles to targeted cells is desirable for selective cell killing. Clusters of gold nanoparticles on T7 phage particles have been fabricated as a PTT delivery platform to treat prostate cancer cells in vitro [206]. The assembly of gold nanoparticles on the capsid surface was achieved via the display of a gold-binding peptide and a prostate cancer cell-binding peptide, in tandem (Figure 5A). Phage-templated gold clusters maintained their cell-targeting functionality and promoted delivery of the system to the cancer cells. Clusters were localized within vesicular organelles (e.g., endosomes), generating even larger clusters with a diameter of up to few hundred nanometers, which suggested receptor-mediated endocytosis as a possible internalization mechanism (Figure 5C,D). Irradiation of the prostate cancer cells resulted in cell death in a very selective manner, whereas no remarkable cell death was observed in both healthy cells and non-targeted cancer cells (Figure 5B).

#### 4.2.5. Incorporation into Polymer Matrices

Polymeric materials are widely used as drug delivery systems because of their ability to be use as a matrix that protects drug molecules and controls drug release via e.g., its rate of degradation or swelling profile. Moreover, in some cases, the high water content and soft structures of polymer matrices make them similar to natural extracellular matrices, which contributes to minimizing tissue irritation and cell adhesion and makes them promising drug delivery systems. However, an initial burst, or very fast release of drug molecules remains a challenge that can limit their use to certain types of drugs. The incorporation of viruses into polymeric matrices has been proposed as a solution to overcome this limitation and to better control the release of the drugs molecules. The affinity-based polymeric drug release system is one such platforms in which M13 phage are embedded inside a polymer matrix to suppress the release of drug proteins due to their specific interactions with phage particles. For this purpose, the p3 coat protein of M13 phage was genetically modified to display peptides that bind to antibodies and mixed with a gelatin solution containing antibodies to form hybrid hydrogels [235]. While the antibodies were gradually released from M13-free gelatin hydrogels within 48 h, their release from the M13-gelatin hybrid hydrogels was ~1% after 144 h, indicating suppression of the release due to phage-antibody interactions.

**Figure 6:**
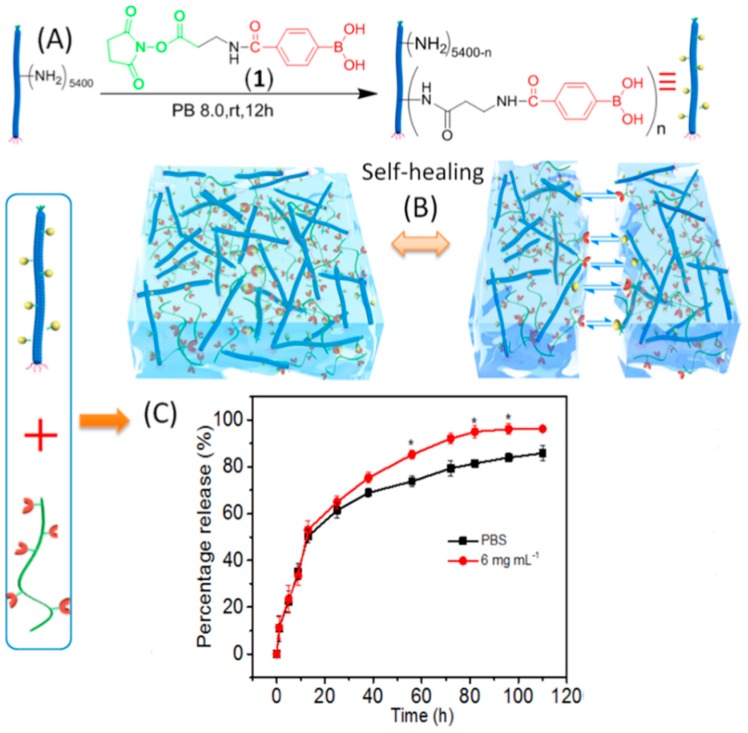
Schematic representing (**A**) the preparation of viral bioconjugates of M13 viruses with low-pKa phenylboronic acid derivative (PBA-M13) and (**B**) fabrication of dynamic hydrogels via binding of PBA-M13 with multiple diol-containing polymers and demonstration of self-healing behavior. (**C**) Glucose responsiveness-regulated insulin release behaviors. FITC-insulin was loaded into the hydrogel and then placed into PBS buffer with or without glucose. The released FITC-insulin was monitored by fluorescent measurements. The asterisks represent the statistical significance, which is calculated by multiple t tests-one per row: * *p* < 0.05, ** *p* < 0.01, *** *p* < 0.001. The hydrogels consist of 1 wt % PBA-M13 and 0.15 wt % PVA (Adapted with permission from [237], Copyright American Chemical Society, 2018).

Stimuli-responsive polymers are another group of material where M13 phage has been introduced to create polymer–protein bioconjugates combining both bioactivities of the protein molecules and the responsive properties of the polymers. A polymer–M13 bioconjugate was produced by grafting a boronic acid containing polymer (poly(NIPAM*co*- phenylboronic acid (PBA)) onto M13 surface through amino groups of the coat proteins [236]. By doing so, around 400 poly(NIPAM*co*-PBA) chains were conjugated per phage particle. Due to the temperature-responsive gelation behavior of the polymer, the polymer–M13 phage bioconjugate was mixed with insulin at 4 ºC and could then be converted into hydrogels by injecting into PBS buffer at 37 ºC. The insulin-encapsulated hydrogels demonstrated glucose-responsive release behavior, which can be an interesting strategy in the regulation of diabetes. During hydrogel formation, the virus particles interact with poly(NIPAMco-PBA) polymer, which is in a collapsed hydrophobic state. However, in the presence of glucose, the boronic acid moieties of poly(NIPAMco-PBA) couple to glucose to yield hydrophilic boronates that are substantially more hydrophilic. This changes the structure of hydrogel matrix and enables faster release of encapsulated insulin molecules. While 70% of insulin was released over 24 h, the release rate of insulin increased in the presence of glucose with a peak value of 90% at 10 h. Similar carbohydrate-responsive polymers have also been prepared by cross-linking phenylboronic acid (PBA)–M13 bioconjugates with poly(vinyl alcohol), resulting in hydrogels with excellent injectability and self-healing behavior (Figure 6A) [237]. The self-healing nature of the hydrogels was investigated by placing two pieces of hydrogels adjacent to each other (Figure 6B). It was observed that the interface became smeared after 15 min and then completely disappeared after 1 h. Additionally, insulin molecules could be introduced into both the PBA–M13 bioconjugate suspension and the PVA solution, resulting in insulin-loaded hydrogels. Glucose-responsive insulin release studies showed that an initial burst release, which was observed in less than 15 h, was similar in the presence and absence of glucose (Figure 6C). At a later stage of release, the hydrogel in the presence of glucose released most of the insulin with a speed faster than that without glucose as a result of swelling of the gel matrix caused by the diffusion of glucose into the hydrogel, which partially disrupts the physical crosslinking between the diols of PVA and the boronic acids.

**Figure 7:**
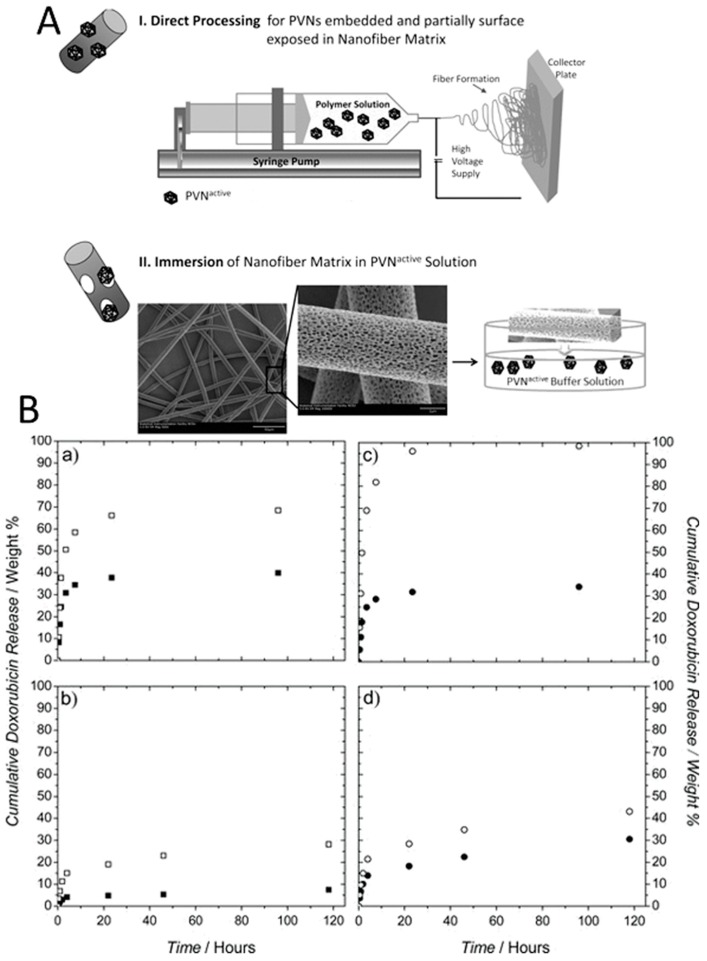
(**A**) Schematic of processes for incorporating plant viral nanoparticles (PVN) in nanofiber matrices. In the direct processing method (I), the PVN active are electrospun in situ with the polymer solution. In the immersion process (II), the nanofiber matrices are dipped into a specific volume and concentration of PVN active particles. (Scale bars of the images from left to right 50.0 μm and 2.0 μm, respectively) (**B**) Cumulative doxorubicin release over time of PLA nanofiber matrices combined with PVN Dox (■) or free doxorubicin (□) where PLA was combined with the active either a) post mat fabrication (dipping method) or b) prior to electrospinning (co-spinning method) and of 70:30 PLA:PEO nanofiber matrices with PVN dox (o) or free doxorubicin (●) where PLA:PEO was combined with the active either c) post mat fabrication (dipping method) or d) prior to electrospinning (co-spinning method) (Adapted with permission from [238], Copyright WILEY-VCH Verlag GmbH & Co., 2013).

RCNMV particles has been another virus incorporated into polymeric drug delivery systems. The ability to trigger the opening and closing of RCNMV capsid pores by changing divalent ion (Ca^2+^ and Mg^2+^) concentrations and pH enables their use as an additional release mechanism in drug delivery systems. Doxorubicin-loaded RCNMV particles could be incorporated into two different polymeric matrices (poly(lactic acid)(PLA) and poly(lactic acid):polyethylene oxide (PLA:PEO)) by electrospinning into fibers [238]. Two approaches were employed to incorporate viral particles in these matrices; i) the direct mixing of viral particles and polymer solution before fiber formation; and ii) physisorption of the viral particles onto the pre-formed fibers by immersion (Figure 7A). It has been demonstrated that electrospinning of drug-loaded viral particles with polymer solutions resulted in nanofibers with significantly lower release profiles (Figure 7B). A slightly lower release profile was also observed for PLA polymer compared to PLA–PEO polymer, which was attributed to the more hydrophilic nature of PEO. Upon appropriate stimuli (reducing divalent ion concentrations or making pH more basic), the release of the drug molecules from virus-polymer matrix, produced via direct processing followed a two-phase kinetic profile. While the first phase included the diffusion of the mobile phase through the polymer to trigger the opening of the virus capsid pores, which enables the release of the drug, in the second phase the drug molecules diffuse through the polymer matrix.

## 5. Challenges to Clinical Applications

Pharmacokinetics and toxicity are critical factors that determine the viability of materials intended to be used as drug delivery system for medical applications. Therefore, several studies investigating the circulation, clearance, blood half-life, stability, immunogenicity, and organ biodistribution of viral platforms have been performed and have provide useful information. While not numerous, some fundamental studies on the interaction of plant viruses and phage with mammalian cells and pathways that they used to enter into cells have been performed. For instance, while M13 phage tends to only bind to the cell membrane of epithelial cells, it shows cell-type dependent interactions and internalization mechanism: clathrin-mediated endocytosis and macropinocytosis for HeLa cells; vesicular transport; clathrin-mediated endocytosis, and macropinocytosis for a human breast cancer cell line (MCF-7); and caveolae-mediated endocytosis for human dermal microvascular endothelial cell (HDMEC) [239]. On the other hand, CPMV particles naturally interact with mammalian cells, including endothelial cells and particularly tumor neovascular endothelium *in vivo*, via a surface exposed cell protein, vimentin [240].

After their administration into the body, viral particles, like many other nanoparticles, are recognized as foreign agents by the cells of the host immune system and are eliminated from the blood. B-cells are one type of these immune cells and B-cell dependent immunoglubins have been shown to play an important role in rapid neutralization of T7 phage in murine blood, resulting in a short half-life (<5 min) [241]. The reticuloendothelial system (RES) is another component of the immune system that is highly involved in the clearance of viral particles. Indeed, clearance by macrophages and accumulation in organs such as the liver and spleen have been reported for many viruses (ca. M13, MS2, CPMV, PVX, and TMV) [22,242,243,244,245]. Nevertheless, there are significant differences between the efficiency of the viral particles in avoiding clearance by phagocytosis, which results in different blood circulation times. For instance, filamentous M13 phage can effectively avoid rapid clearance from the RES with a plasma half-time of 3.6 h [246]. In contrast, another rod-like virus TMV has much shorter blood clearance time (~3 min) [243]. Different plasma clearance half-lives have also been observed for spherical viruses. For instance, while CPMV and CCMV particles are rapidly cleared from the blood with short circulation times (CPMV, 4–7 min), MS2 viral particles show longer plasma half-life [16,208]. It has been demonstrated that certain rod-like particles can effectively evade phagocytosis since the larger contact angles with macrophages do not favor engulfment and internalization. The surface charge of the viral capsid is another parameter that influences their fate in tissues. Due to the negative charge of the surface of mammalian cells resulting from abundant proteoglycans, positively-charged viral particles are expected to interact electrostatically with cells, which has been reported to increase their intracellular delivery and tumor penetration. The short blood circulation of CPMV particles is attributed to their negative surface charge, hence their cellular uptake in HeLa cells could be improved by conjugation of cationic peptide sequences [247].

**Figure 8:**
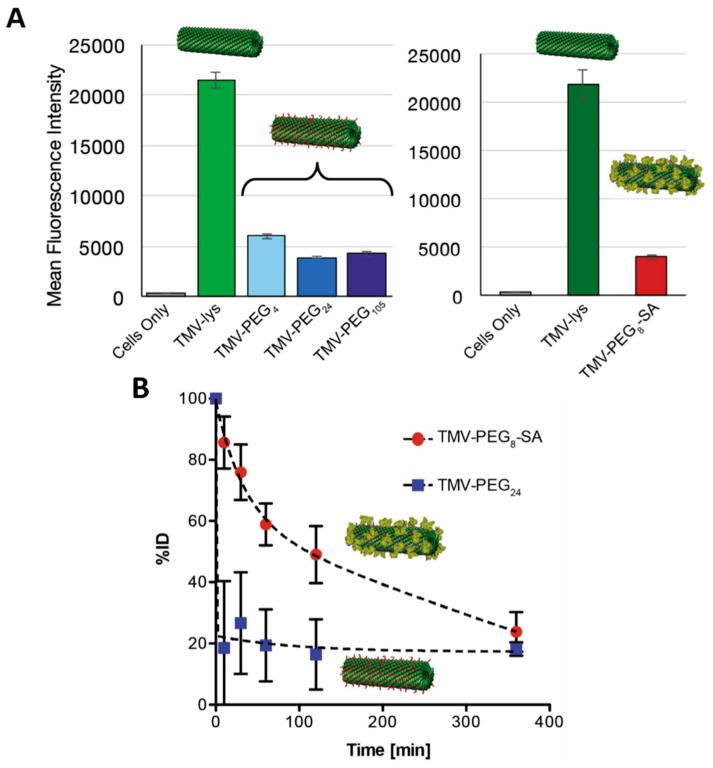
(**A**) In vitro recognition of SA- and PEG-coated TMV vs. ‘naked’ TMV by RAW264.7 macrophages. Quantitative flow cytometry analysis of the interactions between ‘naked’ and ‘stealth’ TMV formulations and RAW264.7 cells. (**B**) Pharmacokinetics of the TMV-PEG24 and TMV-PEG8-SA particles in Balb/C mouse model. The particles were administered intravenously at the amount of 400 mg/mouse. Blood was collected before injection at *t* = 0 and after injection at *t* = 10 min, *t* = 30 min, *t* = 60 min, *t* = 120 min, and *t* = 360 min (the experiments were completed at an *n* = 3 per group) (Adapted with permission from [252], Copyright Elsevier, 2016).

The studies above suggest that the fast blood clearance of viral particles are at least partially due to the host immune response and highlight a need for a coating to tailor the properties of the viral particles for enhanced blood circulation times. For this, the modification of the viral capsid with passivating agents, such as polymers or proteins, has been investigated. For instance, the modification of the CPMV capsid with poly(ethylene glycol) (PEG) effectively blocked CPMV–cell interactions and shielded the viral particles from inducing a primary antibody response [248,249]. The effect of PEGylation has been also observed on plasma circulation times. The half-lives of PEGylated CPMV and TMV particles were 20.8 min and 6.6 min, respectively, which were longer than the half-lives mentioned above [22,250]. In addition to PEG, poly(2-oxazoline) (POx) and serum albumin have also been employed as alternative coatings agents [251]. In comparison to PEGylated TMV, POx-coated viruses showed lower cellular uptake rates indicating less favored TMV–cell interactions due to the higher polymer grafting density of Pox relative to PEG for the tested samples. High polymer density was associated with a reduction in cellular recognition as well as protein adhesion to the polymer-coated particles. On the other hand, pharmacokinetic studies showed a two-phase decay for the polymer-coated TMV particles. During the initial clearance period, POxylated TMV particles had longer plasma half-life (11.3 min) than PEGylated TMV particles (0.01 min) indicating better screening capability of POx polymer. Serum albumin (SA) has been another coating material investigated to shield TMV particles from the immune system. Similar to POx, in cellular uptake and pharmacokinetic studies of the SA coating also outperformed the PEG [252]. While the rates of macrophage uptake of PEG- and SA-coated TMV particles were comparable, showing a 4-fold reduction in macrophage-particle interactions relative to uncoated TMV particles (Figure 8A), their circulation half-lives in Balb/C mice were 10 min and 100 min, respectively (Figure 8B). According to the authors, the enhanced pharmacokinetics of SA coating was associated with its relatively high molecular weight. It was suggested that compared to flexible PEG chains with low molecular weight, the globular and rigid structure of SA provided enhanced steric hindrance, resulting in better stealth properties.

## 6. Conclusions

Overall, a unique feature of viruses highlighted in this review is that they are perfectly-defined nanomaterials that can be conveniently modified, either at the genetic level or using bioconjugate chemistry, to yield interesting tools for drug discovery or drug delivery. Indeed, the structural diversity of viruses as well as the connection between genotype and phenotype has provided several complementary platforms for the discovery of different classes of therapeutic polypeptides by phage display, some of which have made it to the clinic. While in vitro selection procedures by phage display are the most commonplace, a better understanding of the health risks posed by plant viruses and bacteriophages to Humans may unbridle more complex in vivo selection procedures towards targets that cannot be emulated in vitro. Indeed, the available yet limited number of studies examining the application of in vivo phage screening in human patients have shown promising results [253,254]. In contrast to the use of viruses for drug discovery, most studies employing viruses or virus-like particles as drug delivery systems are limited to in vitro proof-of-concepts. Little information is available regarding in vivo toxicity or biodistribution. While some immunohistochemistry studies suggest that virus-like particles do not induce adverse effects in animal tissues, in terms of no overt signs toxicity (tissue degeneration, cell apoptosis, and necrosis), it is necessary to support these results with organ–function studies in the future. This information will be important for guiding future developments of plant and bacterial viruses as drug delivery systems or components thereof. On the other hand, some studies on bacterial viruses has shown that food-ingested phage DNA, like any foreign DNA, can get inside the cells of mouse and, even on rare occasions, can covalently link to mouse DNA [255]. While this information speculates some medically-relevant implications in terms of mutagenesis and carcinogenesis, it has been one of the major concerns preventing the implementation of viral particles in medicine. In this manner, viruses, which enable the removal of genetic material and form empty capsids, could reduce DNA-related concerns and become more interesting as drug carrier platform.

## Figures and Tables

**Table 1:** Plant viruses used in drug delivery systems.

Virus	Size	Symmetry	Family	Genome	Locations on Coat Proteins for Genetic Modification	Ref
**BMV**	30 nm	Icosahedral	*BromoViridae*	ssRNA	Valine 168	[55,56]
**CCMV**	28 nm	Icosahedral	*BromoViridae*	ssRNA	Lysine 42 Serine 102/130	[57,58]
**CPMV**	30 nm	Icosahedral	*Comoviridae*	ssRNA	βB-βC loop of the small subunit/ βE-βF loop of the large subunit	[53,59]
**CMV**	29 nm	Icosahedral	*Bromoviridae*	ssRNA		[60]
**HCRSV**	30 nm	Icosahedral	*Tombusviridae*	ssRNA		[61]
**RCNMV**	36 nm	Icosahedral	*Tombusviridae*	ssRNA		[62]
**TYMV**	28 nm	Icosahedral	*Tymoviridae*	ssRNA	Threonine 44 Lysine 45	[63]
**TMV**	300 × 18 nm	Rod-like	*Tobamoviridae*	ssRNA	Lysine 53/68 Threonine 104/158 Serine 123 N/C-terminal of coat protein	[64,65,66]
**PVX**	515 × 13 nm	Rod-like	*Potexviridae*	ssRNA	N-terminal of coat protein	[67]

**Table 2:** Selected phage display-derived antibodies/peptide therapeutics approved by FDA, as of Dec 06 2018.

Product, Trade Name^®^, Manufacturer, FDA Approval	Type	Target	Phage Display Type	Phage Display Technology	Indication	Ref
**Antibodies**						
Adalimumab (D2E7), Humira^®^, Abbott Laboratories, 2002	IgGκ	TNFα	Humanization by guided selection, scFv	CAT	Rheumatoid arthritis, juvenile idiopathic arthritis, psoriatic arthritis, ankylosing spondylitis, Crohn’s disease, ulcerative colitis, plaque psoriasis	[131,133,147]
Ranibizumab, Lucentis^®^, Genentech, 2006	Fab fragment	VEGF-A	Affinity maturation of bevacizumab by phage display	Genentech	Age-related macular degeneration, macular edema after retinal vein occlusion, diabetic macular edema, diabetic retinopathy	[133,148]
Belimumab, Benlysta^®^, GSK, 2011	IgG1*λ*	BLyS	Naïve, scFv	CAT	Autoantibody-positive, systemic lupus	[133,149,150]
Raxibacumab, Abthrax^®^, GSK, 2012	IgG1*λ*	Protective antigen	Naïve, scFv	CAT	Anthrax	[133,151]
Ramucirumab (IMC-1121B), Cyramza^®^, ImClone/ Lilly, 2014	IgG1	VEGF-R2	Naïve, Fab	Dyax	Gastric cancer, colorectal cancer, non-small cell lung cancer	[133,152]
Necitumumab (IMC-11F8), Portrazza^®^, ImClone/ Lilly, 2015	IgG1*κ*	EGFR	Naïve, Fab	Dyax	Squamous non-small cell lung cancer	[133,153]
Avelumab, Bavencio^®^, EMD Serono/ Pfizer, 2017	IgG1*λ*	PD-L1	Naïve, Fab	Dyax	Metastatic Merkel cell carcinoma	[133,154]
Guselkumab, Tremfya^®^, Janssen, 2017	IgG1*λ*	P19 subunit of Interleukin 23	Synthetic, Fab	MorphoSys	Psoriasis	[133,155]
Lanadelumab (DX-2930), Takhzyro^®^, Shire, 2018	IgG1	Kalikrein	Naïve, Fab	Dyax	Types I and II hereditary angioedema	[133,156,157]
Moxetumomab Pasudotox (HA22 or CAT-8015) Lumoxiti^®^, AstraZeneca, 2018	Antibody-fusion protein	CD22	Affinity maturation of BL22 (CAT-3888) by phage display	CAT	Relapsed or refractory hairy cell leukemia	[158,159]
Emapalumab-lzsg (NI-0501), Gamifant^®^, Novimmune/ Serono, 2018	IgG1	Interferon-gamma	Naïve, scFv	CAT	Primary hemophagocytic lymphohistiocytosis	[133,134]
**Peptides**						
Ecallantide, Kalbitor^®^, Shire, 2009	60 AA polypeptide	Plasma kallikrein	-	Dyax	Hereditary angioedema	[160,161]
Romiplostim, Nplate^®^, Amgen, 2008	Peptide Fc fusion	Thrombopoietin receptor (TPOR)	-	Affymax	Immune thrombocytopenic purpura	[162,163]
Affinity ligand for Xyntha, Wyeth Pharmaceuticals	Polypeptide	Factor VIII	-	Dyax	Hemophilia A	[162,164,165]
Albigutide, Tanzeum^®^, GlaxoSmithKline, 2014	Peptide albumin fusion	GLP-1	-	AlbudAb	Type 2 diabetes mellitus, glycemia	[166,167,168]

AA, amino acid; BLyS, B-lymphocyte stimulator; CAT, Cambridge Antibody Technology; EGFR, epidermal growth factor receptor; FDA, Food and Drug Administration; Fab, fragment antigen-binding; GSK, GlaxoSmithKline; IgG, immunoglobulin G; k, kappa light chain; λ, lambda light chain; PD-L1, programmed death-ligand 1; scFv, single chain fragment variable; VEGFA, vascular endothelial growth factor A; VEGF-R2, vascular endothelial growth factor receptor-2; GLP-1, glucagon-likepeptide-1 receptor; AlbudAb, domain antibodies to serum albumin.

## References

[1] Van der Want J.P.H., Dijkstra J. (2006). A history of plant virology. Arch. Virol..

[2] Brown J.C. (2001). Virology. eLS.

[3] Bos L. (2000). 100 years of virology: From vitalism via molecular biology to genetic engineering. Trends Microbiol..

[4] Kropinski A.M. (2006). Phage Therapy—Everything Old is New Again. Can. J. Infect. Dis. Med. Microbiol..

[5] Merril C.R., Scholl D., Adhya S.L. (2003). The prospect for bacteriophage therapy in Western medicine. Nat. Rev. Drug Discov..

[6] Sunderland K.S., Yang M., Mao C. (2017). Phage-Enabled Nanomedicine: From Probes to Therapeutics in Precision Medicine. Angew. Chem..

[7] Henry M., Debarbieux L. (2012). Tools from viruses: Bacteriophage successes and beyond. Virology.

[8] Domingo-Calap P., Georgel P., Bahram S. (2016). Back to the future: Bacteriophages as promising therapeutic tools. HLA.

[9] Méthot P.-O. (2016). Writing the history of virology in the twentieth century: Discovery, disciplines, and conceptual change. Stud. Hist. Philos. Sci. Part C.

[10] The Royal Swedish Academy of Sciences Press Release: The Nobel Prize in Chemistry 2018. https://www.nobelprize.org/prizes/chemistry/2018/press-release/.

[11] Nayerossadat N., Maedeh T., Ali P.A. (2012). Viral and nonviral delivery systems for gene delivery. Adv. Biomed. Res..

[12] Lundstrom K. (2018). Viral Vectors in Gene Therapy. Diseases.

[13] Liu Z., Qiao J., Niu Z., Wang Q. (2012). Natural supramolecular building blocks: From virus coat proteins to viral nanoparticles. Chem. Soc. Rev..

[14] Pokorski J.K., Steinmetz N.F. (2011). The Art of Engineering Viral Nanoparticles. Mol. Pharm..

[15] Kaiser C.R., Flenniken M.L., Gillitzer E., Harmsen A.L., Harmsen A.G., Jutila M.A., Douglas T., Young M.J. (2007). Biodistribution studies of protein cage nanoparticles demonstrate broad tissue distribution and rapid clearance in vivo. Int. J. Nanomed..

[16] Singh P., Prasuhn D., Yeh R.M., Destito G., Rae C.S., Osborn K., Finn M.G., Manchester M. (2007). Bio-distribution, toxicity and pathology of cowpea mosaic virus nanoparticles in vivo. J. Control. Release.

[17] Green N.K., Herbert C.W., Hale S.J., Hale A.B., Mautner V., Harkins R., Hermiston T., Ulbrich K., Fisher K.D., Seymour L.W. (2004). Extended plasma circulation time and decreased toxicity of polymer-coated adenovirus. Gene Ther..

[18] Vaks L., Benhar I. (2011). In vivo characteristics of targeted drug-carrying filamentous bacteriophage nanomedicines. J. Nanobiotechnol..

[19] Flynn C.E., Lee S.-W., Peelle B.R., Belcher A.M. (2003). Viruses as vehicles for growth, organization and assembly of materials11The Golden Jubilee Issue—Selected topics in Materials Science and Engineering: Past, Present and Future, edited by *S. Suresh*. Acta Mater..

[20] Lee S.-Y., Lim J.-S., Harris M.T. (2012). Synthesis and application of virus-based hybrid nanomaterials. Biotechnol. Bioeng..

[21] Wen A.M., Wang Y., Jiang K., Hsu G.C., Gao H., Lee K.L., Yang A.C., Yu X., Simon D.I., Steinmetz N.F. (2015). Shaping bio-inspired nanotechnologies to target thrombosis for dual optical-magnetic resonance imaging. J. Mater. Chem. B.

[22] Shukla S., Ablack A.L., Wen A.M., Lee K.L., Lewis J.D., Steinmetz N.F. (2013). Increased Tumor Homing and Tissue Penetration of the Filamentous Plant Viral Nanoparticle Potato virus X. Mol. Pharm..

[23] Rong J., Niu Z., Lee L.A., Wang Q. (2011). Self-assembly of viral particles. Curr. Opin. Colloid Interface Sci..

[24] Russel M., Lowman H.B., Clackson T., Clackson T., Lowman H.B. (2004). Introduction to Phage Biology and Phage Display. Phage Display: A Practical Approach.

[25] Hemminga M.A., Vos W.L., Nazarov P.V., Koehorst R.B.M., Wolfs C.J.A.M., Spruijt R.B., Stopar D. (2010). Viruses: Incredible nanomachines. New advances with filamentous phages. Eur. Biophys. J..

[26] Kehoe J.W., Kay B.K. (2005). Filamentous Phage Display in the New Millennium. Chem. Rev..

[27] Sidhu S.S. (2001). Engineering M13 for phage display. Biomol. Eng..

[28] Clokie M.R.J., Millard A.D., Letarov A.V., Heaphy S. (2011). Phages in nature. Bacteriophage.

[29] Salmond G.P.C., Fineran P.C. (2015). A century of the phage: Past, present and future. Nat. Rev. Microbiol..

[30] Onodera K., Endo I., Nagamune T. (2010). Molecular Biology and Biotechnology of Bacteriophage. Nano/Micro Biotechnology.

[31] Tzagoloff H., Pratt D. (1964). The initial steps in infection with coliphage M13. Virology.

[32] Kurzępa A., Dąbrowska K., Świtała-Jeleń K., Górski A. (2009). Molecular modification of T4 bacteriophage proteins and its potential application—Review. Folia Microbiol..

[33] Sathaliyawala T., Islam M.Z., Li Q., Fokine A., Rossmann M.G., Rao V.B. (2010). Functional Analysis of the Highly Antigenic Outer Capsid Protein, Hoc, a Virus Decoration Protein from T4-like Bacteriophages. Mol. Microbiol..

[34] Sathaliyawala T., Rao M., Maclean D.M., Birx D.L., Alving C.R., Rao V.B. (2006). Assembly of Human Immunodeficiency Virus (HIV) Antigens on Bacteriophage T4: A Novel In Vitro Approach To Construct Multicomponent HIV Vaccines. J. Virol..

[35] Fokine A., Bowman V.D., Battisti A.J., Li Q., Chipman P.R., Rao V.B., Rossmann M.G. (2007). Cryo-electron microscopy study of bacteriophage T4 displaying anthrax toxin proteins. Virology.

[36] Kemp C.L., Howatson A.F., Siminovitch L. (1968). Electron microscope studies of mutants of lambda bacteriophage: I. General description and quantitation of viral products. Virology.

[37] Nguyen T.H., Easter N., Gutierrez L., Huyett L., Defnet E., Mylon S.E., Ferri J.K., Viet N.A. (2011). The RNA core weakly influences the interactions of the bacteriophage MS2 at key environmental interfaces. Soft Matter.

[38] Carrillo-Tripp M., Shepherd C.M., Borelli I.A., Venkataraman S., Lander G., Natarajan P., Johnson J.E., Brooks C.L., Reddy V.S. (2009). VIPERdb2: An enhanced and web API enabled relational database for structural virology. Nucleic Acids Res..

[39] Doan D.N.P., Lee K.C., Laurinmäki P., Butcher S., Wong S.M., Dokland T. (2003). Three-dimensional reconstruction of hibiscus chlorotic ringspot virus. J. Struct. Biol..

[40] Sachse C., Chen J.Z., Coureux P.D., Stroupe M.E., Fändrich M., Grigorieff N. (2007). High-resolution Electron Microscopy of Helical Specimens: A Fresh Look at Tobacco Mosaic Virus. J. Mol. Biol..

[41] Le D.H.T., Hu H., Commandeur U., Steinmetz N.F. (2017). Chemical addressability of potato virus X for its applications in bio/nanotechnology. J. Struct. Biol..

[42] Agirrezabala X., Martín-Benito J., Castón J.R., Miranda R., Valpuesta J.M., Carrascosa J.L. (2005). Maturation of phage T7 involves structural modification of both shell and inner core components. EMBO J..

[43] Cerritelli M.E., Studier W.F. (1996). Assembly of T7 Capsids from Independently Expressed and Purified Head Protein and Scaffolding Protein. J. Mol. Biol..

[44] Fuller D.N., Raymer D.M., Rickgauer J.P., Robertson R.M., Catalano C.E., Anderson D.L., Grimes S., Smith D.E. (2007). Measurements of single DNA molecule packaging dynamics in bacteriophage lambda reveal high forces, high motor processivity, and capsid transformations. J. Mol. Biol..

[45] Lander G.C., Evilevitch A., Jeembaeva M., Potter C.S., Carragher B., Johnson J.E. (2008). Bacteriophage lambda stabilization by auxiliary protein gpD: Timing, location, and mechanism of attachment determined by cryo-EM. Structure.

[46] Valegård K., Liljas L., Fridborg K., Unge T. (1990). The three-dimensional structure of the bacterial virus MS2. Nature.

[47] Fu Y., Li J. (2016). A novel delivery platform based on Bacteriophage MS2 virus-like particles. Virus Res..

[48] Ma Y., Nolte R.J.M., Cornelissen J.J.L.M. (2012). Virus-based nanocarriers for drug delivery. Adv. Drug Deliv. Rev..

[49] Hooker J.M., Kovacs E.W., Francis M.B. (2004). Interior Surface Modification of Bacteriophage MS2. J. Am. Chem. Soc..

[50] Wen A.M., Steinmetz N.F. (2016). Design of virus-based nanomaterials for medicine, biotechnology, and energy. Chem. Soc. Rev..

[51] Barnhill H.N., Reuther R., Ferguson P.L., Dreher T., Wang Q. (2007). Turnip Yellow Mosaic Virus as a Chemoaddressable Bionanoparticle. Bioconj. Chem..

[52] Wang Q., Lin T., Tang L., Johnson J.E., Finn M.G. (2002). Icosahedral Virus Particles as Addressable Nanoscale Building Blocks. Angew. Chem. Int. Ed..

[53] Wang Q., Kaltgrad E., Lin T., Johnson J.E., Finn M.G. (2002). Natural Supramolecular Building Blocks: Wild-Type Cowpea Mosaic Virus. Chem. Biol..

[54] Narayanan K.B., Han S.S. (2017). Icosahedral plant viral nanoparticles—Bioinspired synthesis of nanomaterials/nanostructures. Adv. Colloid Interface Sci..

[55] Yildiz I., Tsvetkova I., Wen A.M., Shukla S., Masarapu M.H., Dragnea B., Steinmetz N.F. (2012). Engineering of Brome mosaic virus for biomedical applications. RSC Adv..

[56] Dixit S.K., Goicochea N.L., Daniel M.-C., Murali A., Bronstein L., De M., Stein B., Rotello V.M., Kao C.C., Dragnea B. (2006). Quantum Dot Encapsulation in Viral Capsids. Nano Lett..

[57] Suci P.A., Varpness Z., Gillitzer E., Douglas T., Young M. (2007). Targeting and Photodynamic Killing of a Microbial Pathogen Using Protein Cage Architectures Functionalized with a Photosensitizer. Langmuir.

[58] Zlotnick A., Aldrich R., Johnson J.M., Ceres P., Young M.J. (2000). Mechanism of Capsid Assembly for an Icosahedral Plant Virus. Virology.

[59] Huynh N.T., Hesketh E.L., Saxena P., Meshcheriakova Y., Ku Y.-C., Hoang L.T., Johnson J.E., Ranson N.A., Lomonossoff G.P., Reddy V.S. (2016). Crystal Structure and Proteomics Analysis of Empty Virus-like Particles of Cowpea Mosaic Virus. Structure.

[60] Zeng Q., Wen H., Wen Q., Chen X., Wang Y., Xuan W., Liang J., Wan S. (2013). Cucumber mosaic virus as drug delivery vehicle for doxorubicin. Biomaterials.

[61] Ren Y., Wong S.-M., Lim L.-Y. (2006). In vitro-reassembled plant virus-like particles for loading of polyacids. J. Gen. Virol..

[62] Lockney D.M., Guenther R.N., Loo L., Overton W., Antonelli R., Clark J., Hu M., Luft C., Lommel S.A., Franzen S. (2011). The Red clover necrotic mosaic virus Capsid as a Multifunctional Cell Targeting Plant Viral Nanoparticle. Bioconj. Chem..

[63] Dreher T.W. (2004). Turnip yellow mosaic virus: Transfer RNA mimicry, chloroplasts and a C-rich genome. Mol. Plant Pathol..

[64] Finbloom J.A., Han K., Aanei I.L., Hartman E.C., Finley D.T., Dedeo M.T., Fishman M., Downing K.H., Francis M.B. (2016). Stable Disk Assemblies of a Tobacco Mosaic Virus Mutant as Nanoscale Scaffolds for Applications in Drug Delivery. Bioconj. Chem..

[65] Shukla S., Eber F.J., Nagarajan A.S., DiFranco N.A., Schmidt N., Wen A.M., Eiben S., Twyman R.M., Wege C., Steinmetz N.F. (2015). The Impact of Aspect Ratio on the Biodistribution and Tumor Homing of Rigid Soft-Matter Nanorods. Adv. Healthc. Mater..

[66] Bazzini A.A., Hopp H.E., Beachy R.N., Asurmendi S. (2007). Infection and coaccumulation of tobacco mosaic virus proteins alter microRNA levels, correlating with symptom and plant development. Proc. Natl. Acad. Sci. USA.

[67] Shukla S., Dickmeis C., Nagarajan A.S., Fischer R., Commandeur U., Steinmetz N.F. (2014). Molecular farming of fluorescent virus-based nanoparticles for optical imaging in plants, human cells and mouse models. Biomater. Sci..

[68] Kwak M., Minten I.J., Anaya D.-M., Musser A.J., Brasch M., Nolte R.J.M., Müllen K., Cornelissen J.J.L.M., Herrmann A. (2010). Virus-like Particles Templated by DNA Micelles: A General Method for Loading Virus Nanocarriers. J. Am. Chem. Soc..

[69] Saunders K., Sainsbury F., Lomonossoff G.P. (2009). Efficient generation of cowpea mosaicvirus empty virus-like particles by the proteolytic processing of precursors in insect cells and plants. Virology.

[70] Marín-Caba L., Chariou P.L., Pesquera C., Correa-Duarte M.A., Steinmetz N.F. (2019). Tobacco Mosaic Virus-Functionalized Mesoporous Silica Nanoparticles, a Wool-Ball-like Nanostructure for Drug Delivery. Langmuir.

[71] Bruckman M.A., Hern S., Jiang K., Flask C.A., Yu X., Steinmetz N.F. (2013). Tobacco mosaic virus rods and spheres as supramolecular high-relaxivity MRI contrast agents. J. Mater. Chem. B.

[72] Niehl A., Appaix F., Boscá S., van der Sanden B., Nicoud J.-F., Bolze F., Heinlein M. (2016). Fluorescent Tobacco mosaic virus-Derived Bio-Nanoparticles for Intravital Two-Photon Imaging. Front. Plant Sci..

[73] Sánchez F., Sáez M., Lunello P., Ponz F. (2013). Plant viral elongated nanoparticles modified for log-increases of foreign peptide immunogenicity and specific antibody detection. J. Biotechnol..

[74] Smith G.P. (1985). Filamentous fusion phage: Novel expression vectors that display cloned antigens on the virion surface. Science.

[75] Huang J.X., Bishop-Hurley S.L., Cooper M.A. (2012). Development of Anti-Infectives Using Phage Display: Biological Agents against Bacteria, Viruses, and Parasites. Antimicrob. Agents Chemother..

[76] Seker U.O.S., Demir H.V. (2011). Material Binding Peptides for Nanotechnology. Molecules.

[77] Merzlyak A., Lee S.-W. (2006). Phage as templates for hybrid materials and mediators for nanomaterial synthesis. Curr. Opin. Chem. Biol..

[78] Kriplani U., Kay B.K. (2005). Selecting peptides for use in nanoscale materials using phage-displayed combinatorial peptide libraries. Curr. Opin. Biotechnol..

[79] Garet E., Cabado A.G., Vieites J.M., González-Fernández Á. (2010). Rapid isolation of single-chain antibodies by phage display technology directed against one of the most potent marine toxins: Palytoxin. Toxicon.

[80] Nanduri V., Bhunia A.K., Tu S.-I., Paoli G.C., Brewster J.D. (2007). SPR biosensor for the detection of L. monocytogenes using phage-displayed antibody. Biosens. Bioelectron..

[81] Pasqualini R., Ruoslahti E. (1996). Organ targeting In vivo using phage display peptide libraries. Nature.

[82] Deutscher S.L. (2010). Phage Display in Molecular Imaging and Diagnosis of Cancer. Chem. Rev..

[83] Govarts C., Somers K., Stinissen P., Somers V. (2010). Frameshifting in the P6 cDNA Phage Display System. Molecules.

[84] Ghosh D., Kohli A.G., Moser F., Endy D., Belcher A.M. (2012). Refactored M13 Bacteriophage as a Platform for Tumor Cell Imaging and Drug Delivery. ACS Synth. Biol..

[85] Gao C., Mao S., Lo C.-H.L., Wirsching P., Lerner R.A., Janda K.D. (1999). Making artificial antibodies: A format for phage display of combinatorial heterodimeric arrays. Proc. Natl. Acad. Sci. USA.

[86] Pande J., Szewczyk M.M., Grover A.K. (2010). Phage display: Concept, innovations, applications and future. Biotechnol. Adv..

[87] Makowski L. (1994). Phage display: Structure, assembly and engineering of filamentous bacteriophage M13. Curr. Opin. Struct. Biol..

[88] Smith G.P. (1993). Preface. Gene.

[89] Molek P., Bratkovič T. (2015). Bacteriophages as Scaffolds for Bipartite Display: Designing Swiss Army Knives on a Nanoscale. Bioconjugate Chemistry.

[90] Morton V.L., Burkitt W., O’Connor G., Stonehouse N.J., Stockley P.G., Ashcroft A.E. (2010). RNA-induced conformational changes in a viral coat protein studied by hydrogen/deuterium exchange mass spectrometry. Phys. Chem. Chem. Phys..

[91] Wilson D.R., Finlay B.B. (1998). Phage display: Applications, innovations, and issues in phage and host biology. Can. J. Microbiol..

[92] Smith G.P., Petrenko V.A. (1997). Phage Display. Chem. Rev..

[93] Ren Z.J., Black L.W., Lewis G.K., Wingfield P.T., Locke E.G., Steven A.C. (1996). Phage display of intact domains at high copy number: A system based on SOC, the small outer capsid protein of bacteriophage T4. Protein Sci..

[94] Skerra A., Plückthun A. (1991). Secretion and in vivo folding of the Fab fragment of the antibody McPC603 in Escherichia coli: Influence of disulphides and cis-prolines. Protein Eng..

[95] Bratkovič T. (2010). Progress in phage display: Evolution of the technique and its applications. Cell. Mol. Life Sci..

[96] Gao J., Wang Y., Liu Z., Wang Z. (2010). Phage display and its application in vaccine design. Ann. Microbiol..

[97] Wu J., Tu C., Yu X., Zhang M., Zhang N., Zhao M., Nie W., Ren Z. (2007). Bacteriophage T4 nanoparticle capsid surface SOC and HOC bipartite display with enhanced classical swine fever virus immunogenicity: A powerful immunological approach. J. Virol. Methods.

[98] Ren Z.-j., Black L.W. (1998). Phage T4 SOC and HOC display of biologically active, full-length proteins on the viral capsid. Gene.

[99] Oślizło A., Miernikiewicz P., Piotrowicz A., Owczarek B., Kopciuch A., Figura G., Dąbrowska K. (2011). Purification of phage display-modified bacteriophage T4 by affinity chromatography. BMC Biotechnol..

[100] Jiang J., Abu-Shilbayeh L., Rao V.B. (1997). Display of a PorA peptide from Neisseria meningitidis on the bacteriophage T4 capsid surface. Infect. Immun..

[101] Li Q., Shivachandra S.B., Zhang Z., Rao V.B. (2007). Assembly of the Small Outer Capsid Protein, Soc, on Bacteriophage T4: A novel system for high density display of multiple large anthrax toxins and foreign proteins on phage capsid. J. Mol. Biol..

[102] Rao V.B., Black L.W. (2010). Structure and assembly of bacteriophage T4 head. Virol. J..

[103] Gamkrelidze M., Dąbrowska K. (2014). T4 bacteriophage as a phage display platform. Arch. Microbiol..

[104] Danner S., Belasco J.G. (2001). T7 phage display: A novel genetic selection system for cloning RNA-binding proteins from cDNA libraries. Proc. Natl. Acad. Sci. USA.

[105] Krumpe L.R.H., Atkinson A.J., Smythers G.W., Kandel A., Schumacher K.M., McMahon J.B., Makowski L., Mori T. (2006). T7 lytic phage-displayed peptide libraries exhibit less sequence bias than M13 filamentous phage-displayed peptide libraries. Proteomics.

[106] Caberoy N.B., Zhou Y., Jiang X., Alvarado G., Li W. (2010). Efficient identification of tubby-binding proteins by an improved system of T7 phage display. J. Mol. Recognit..

[107] Sharma S.C., Memic A., Rupasinghe C.N., Duc A.-C.E., Spaller M.R. (2009). T7 phage display as a method of peptide ligand discovery for PDZ domain proteins. Pept. Sci..

[108] Maruyama I.N., Maruyama H.I., Brenner S. (1994). Lambda foo: A lambda phage vector for the expression of foreign proteins. Proc. Natl. Acad. Sci. USA.

[109] Gi Mikawa Y., Maruyama I.N., Brenner S. (1996). Surface Display of Proteins on Bacteriophage λ Heads. J. Mol. Biol..

[110] Hoess R.H. (2002). Bacteriophage Lambda as a Vehicle for Peptide and Protein Display. Curr. Pharm. Biotechnol..

[111] Zanghi C.N., Lankes H.A., Bradel-Tretheway B., Wegman J., Dewhurst S. (2005). A simple method for displaying recalcitrant proteins on the surface of bacteriophage lambda. Nucleic Acids Res..

[112] Beghetto E., Gargano N. (2011). Lambda-Display: A Powerful Tool for Antigen Discovery. Molecules.

[113] Nicastro J., Sheldon K., Slavcev R.A. (2014). Bacteriophage lambda display systems: Developments and applications. Appl. Microbiol. Biotechnol..

[114] Heal K.G., Hill H.R., Stockley P.G., Hollingdale M.R., Taylor-Robinson A.W. (1999). Expression and immunogenicity of a liver stage malaria epitope presented as a foreign peptide on the surface of RNA-free MS2 bacteriophage capsids. Vaccine.

[115] Peabody D.S., Manifold-Wheeler B., Medford A., Jordan S.K., do Carmo Caldeira J., Chackerian B. (2008). Immunogenic Display of Diverse Peptides on Virus-like Particles of RNA Phage MS2. J. Mol. Biol..

[116] Mastico R.A., Talbot S.J., Stockley P.G. (1993). Multiple presentation of foreign peptides on the surface of an RNA-free spherical bacteriophage capsid. J. Gen. Virol..

[117] Chackerian B., Caldeira J.d.C., Peabody J., Peabody D.S. (2011). Peptide Epitope Identification by Affinity Selection on Bacteriophage MS2 Virus-Like Particles. J. Mol. Biol..

[118] Brown W.L., Mastico R.A., Wu M., Heal K.G., Adams C.J., Murray J.B., Simpson J.C., Lord J.M., Taylor-Robinson A.W., Stockley P.G. (2002). RNA Bacteriophage Capsid-Mediated Drug Delivery and Epitope Presentation. Intervirology.

[119] Peabody D.S. (1997). Subunit Fusion Confers Tolerance to Peptide Insertions in a Virus Coat Protein. Arch. Biochem. Biophys..

[120] Van Meerten D., Olsthoorn R.C.L., van Duin J., Verhaert R.M.D. (2001). Peptide display on live MS2 phage: Restrictions at the RNA genome level. J. Gen. Virol..

[121] Nixon A.E. (2002). Phage display as a tool for protease ligand discovery. Curr. Pharm. Biotechnol..

[122] Fernandez-Gacio A., Uguen M., Fastrez J. (2003). Phage display as a tool for the directed evolution of enzymes. Trends Biotechnol..

[123] Li B., Tom J.Y., Oare D., Yen R., Fairbrother W.J., Wells J.A., Cunningham B.C. (1995). Minimization of a polypeptide hormone. Science.

[124] Kronqvist N., Malm M., Gostring L., Gunneriusson E., Nilsson M., Hoiden Guthenberg I., Gedda L., Frejd F.Y., Stahl S., Lofblom J. (2011). Combining phage and staphylococcal surface display for generation of ErbB3-specific Affibody molecules. Protein Eng. Des. Sel..

[125] Rader C., Cheresh D.A., Barbas C.F. (1998). A phage display approach for rapid antibody humanization: Designed combinatorial V gene libraries. Proc. Natl. Acad. Sci. USA.

[126] Kohler G., Milstein C. (1975). Continuous cultures of fused cells secreting antibody of predefined specificity. Nature.

[127] Emmons C., Hunsicker L.G. (1987). Muromonab-CD3 (Orthoclone OKT3): The first monoclonal antibody approved for therapeutic use. IOWA Med..

[128] Hwang W.Y.K., Foote J. (2005). Immunogenicity of engineered antibodies. Methods.

[129] Ishida I., Tomizuka K., Yoshida H., Tahara T., Takahashi N., Ohguma A., Tanaka S., Umehashi M., Maeda H., Nozaki C. (2002). Production of Human Monoclonal and Polyclonal Antibodies in TransChromo Animals. Cloning Stem Cells.

[130] Ma B., Osborn M.J., Avis S., Ouisse L.-H., Ménoret S., Anegon I., Buelow R., Brüggemann M. (2013). Human antibody expression in transgenic rats: Comparison of chimeric IgH loci with human VH, D and JH but bearing different rat C-gene regions. J. Immunol. Methods.

[131] Nelson A.L., Dhimolea E., Reichert J.M. (2010). Development trends for human monoclonal antibody therapeutics. Nat. Rev. Drug Discov..

[132] Tsuruta L.R., Lopes dos M., Ana Maria Moro A.M., Böldicke T. (2017). Display Technologies for the Selection of Monoclonal Antibodies for Clinical Use. Antibody Engineering.

[133] Frenzel A., Schirrmann T., Hust M. (2016). Phage display-derived human antibodies in clinical development and therapy. MAbs.

[134] Kaplon H., Reichert J.M. (2018). Antibodies to watch in 2019. MAbs.

[135] Hust M., Meyer T., Voedisch B., Rülker T., Thie H., El-Ghezal A., Kirsch M.I., Schütte M., Helmsing S., Meier D. (2011). A human scFv antibody generation pipeline for proteome research. J. Biotechnol..

[136] Schofield D.J., Pope A.R., Clementel V., Buckell J., Chapple S.D., Clarke K.F., Conquer J.S., Crofts A.M., Crowther S.R.E., Dyson M.R. (2007). Application of phage display to high throughput antibody generation and characterization. Genome Biol..

[137] De Haard H.J., van Neer N., Reurs A., Hufton S.E., Roovers R.C., Henderikx P., de Bruïne A.P., Arends J.-W., Hoogenboom H.R. (1999). A Large Non-immunized Human Fab Fragment Phage Library That Permits Rapid Isolation and Kinetic Analysis of High Affinity Antibodies. J. Biol. Chem..

[138] Hoet R.M., Cohen E.H., Kent R.B., Rookey K., Schoonbroodt S., Hogan S., Rem L., Frans N., Daukandt M., Pieters H. (2005). Generation of high-affinity human antibodies by combining donor-derived and synthetic complementarity-determining-region diversity. Nat. Biotechnol..

[139] Mazor Y., Van Blarcom T., Carroll S., Georgiou G. (2010). Selection of full-length IgGs by tandem display on filamentous phage particles and *Escherichia coli* fluorescence-activated cell sorting screening. FEBS J..

[140] Hoogenboom H.R. (2005). Selecting and screening recombinant antibody libraries. Nat. Biotechnol..

[141] Kennedy P.J., Oliveira C., Granja P.L., Sarmento B. (2018). Monoclonal antibodies: Technologies for early discovery and engineering. Crit. Rev. Biotechnol..

[142] Vaughan T.J., Williams A.J., Pritchard K., Osbourn J.K., Pope A.R., Earnshaw J.C., McCafferty J., Hodits R.A., Wilton J., Johnson K.S. (1996). Human Antibodies with Sub-nanomolar Affinities Isolated from a Large Non-immunized Phage Display Library. Nat. Biotechnol..

[143] Lloyd C., Lowe D., Edwards B., Welsh F., Dilks T., Hardman C., Vaughan T. (2008). Modelling the human immune response: Performance of a 1011 human antibody repertoire against a broad panel of therapeutically relevant antigens. Protein Eng. Des. Sel..

[144] Kügler J., Wilke S., Meier D., Tomszak F., Frenzel A., Schirrmann T., Dübel S., Garritsen H., Hock B., Toleikis L. (2015). Generation and analysis of the improved human HAL9/10 antibody phage display libraries. BMC Biotechnol..

[145] Nelson B., Sidhu S.S., Voynov V., Caravella J.A. (2012). Synthetic Antibody Libraries. Therapeutic Proteins: Methods and Protocols.

[146] Osbourn J., Groves M., Vaughan T. (2005). From rodent reagents to human therapeutics using antibody guided selection. Methods.

[147] Burmester G.R., Panaccione R., Gordon K.B., McIlraith M.J., Lacerda A.P. (2013). Adalimumab: Long-term safety in 23 458 patients from global clinical trials in rheumatoid arthritis, juvenile idiopathic arthritis, ankylosing spondylitis, psoriatic arthritis, psoriasis and Crohn’s disease. Ann. Rheum. Dis..

[148] Group C.R., Martin D.F., Maguire M.G., Ying G.S., Grunwald J.E., Fine S.L., Jaffe G.J. (2011). Ranibizumab and bevacizumab for neovascular age-related macular degeneration. N. Engl. J. Med..

[149] Stohl W., Hilbert D.M. (2012). The discovery and development of belimumab: The anti-BLyS-lupus connection. Nat. Biotechnol..

[150] Navarra S.V., Guzman R.M., Gallacher A.E., Hall S., Levy R.A., Jimenez R.E., Li E.K., Thomas M., Kim H.Y., Leon M.G. (2011). Efficacy and safety of belimumab in patients with active systemic lupus erythematosus: A randomised, placebo-controlled, phase 3 trial. Lancet.

[151] Mazumdar S. (2009). Raxibacumab. MAbs.

[152] Aprile G., Bonotto M., Ongaro E., Pozzo C., Giuliani F. (2013). Critical appraisal of ramucirumab (IMC-1121B) for cancer treatment: From benchside to clinical use. Drugs.

[153] Dienstmann R., Felip E. (2011). Necitumumab in the treatment of advanced non-small cell lung cancer: Translation from preclinical to clinical development. Expert Opin. Biol..

[154] Chin K., Chand V.K., Nuyten D.S.A. (2017). Avelumab: Clinical trial innovation and collaboration to advance anti-PD-L1 immunotherapy. Ann. Oncol..

[155] Machado A., Torres T. (2018). Guselkumab for the Treatment of Psoriasis. BioDrugs.

[156] Kenniston J.A., Faucette R.R., Martik D., Comeau S.R., Lindberg A.P., Kopacz K.J., Conley G.P., Chen J., Viswanathan M., Kastrapeli N. (2014). Inhibition of plasma kallikrein by a highly specific active site blocking antibody. J. Biol. Chem..

[157] Banerji A., Busse P., Shennak M., Lumry W., Davis-Lorton M., Wedner H.J., Jacobs J., Baker J., Bernstein J.A., Lockey R. (2017). Inhibiting Plasma Kallikrein for Hereditary Angioedema Prophylaxis. N. Engl. J. Med..

[158] Kreitman R.J., Pastan I. (2011). Antibody fusion proteins: Anti-CD22 recombinant immunotoxin moxetumomab pasudotox. Clin. Cancer Res..

[159] Kreitman R.J., Dearden C., Zinzani P.L., Delgado J., Karlin L., Robak T., Gladstone D.E., le Coutre P., Dietrich S., Gotic M. (2018). Moxetumomab pasudotox in relapsed/refractory hairy cell leukemia. Leukemia.

[160] Markland W., Ley A.C., Ladner R.C. (1996). Iterative optimization of high-affinity protease inhibitors using phage display. 2. Plasma kallikrein and thrombin. Biochemistry.

[161] Farkas H., Varga L. (2011). Ecallantide is a novel treatment for attacks of hereditary angioedema due to C1 inhibitor deficiency. Clin. Cosmet. Investig. Derm..

[162] Nixon A.E., Sexton D.J., Ladner R.C. (2014). Drugs derived from phage display: From candidate identification to clinical practice. MAbs.

[163] Molineux G., Newland A. (2010). Development of romiplostim for the treatment of patients with chronic immune thrombocytopenia: From bench to bedside. Br. J. Haematol..

[164] Kelley B.D., Booth J., Tannatt M., Wub Q.L., Ladner R., Yuc J., Potter D., Ley A. (2004). Isolation of a peptide ligand for affinity purification of factor VIII using phage display. J. Chromatogr. A.

[165] Kelley B., Jankowski M., Booth J. (2010). An improved manufacturing process for Xyntha/ReFacto AF. Haemophilia.

[166] Mimmi S., Maisano D., Quinto I., Iaccino E. (2019). Phage Display: An Overview in Context to Drug Discovery. Trends Pharmacol. Sci..

[167] Fala L. (2015). Tanzeum (Albiglutide): A Once-Weekly GLP-1 Receptor Agonist Subcutaneous Injection Approved for the Treatment of Patients with Type 2 Diabetes. Am. Health Drug Benefits.

[168] Bao W., Holt L.J., Prince R.D., Jones G.X., Aravindhan K., Szapacs M., Barbour A.M., Jolivette L.J., Lepore J.J., Willette R.N. (2013). Novel fusion of GLP-1 with a domain antibody to serum albumin prolongs protection against myocardial ischemia/reperfusion injury in the rat. Cardiovasc. Diabetol..

[169] Wilson P.C., Andrews S.F. (2012). Tools to therapeutically harness the human antibody response. Nat. Rev. Immunol..

[170] Brown S. (1997). Metal-recognition by repeating polypeptides. Nat. Biotechnol..

[171] Brown S. (1992). Engineered iron oxide-adhesion mutants of the Escherichia coli phage lambda receptor. Proc. Natl. Acad. Sci. USA.

[172] Whaley S.R., English D.S., Hu E.L., Barbara P.F., Belcher A.M. (2000). Selection of peptides with semiconductor binding specificity for directed nanocrystal assembly. Nature.

[173] Seker U.O.S., Wilson B., Dincer S., Kim I.W., Oren E.E., Evans J.S., Tamerler C., Sarikaya M. (2007). Adsorption Behavior of Linear and Cyclic Genetically Engineered Platinum Binding Peptides. Langmuir.

[174] Sanghvi A.B., Miller K.P.H., Belcher A.M., Schmidt C.E. (2005). Biomaterials functionalization using a novel peptide that selectively binds to a conducting polymer. Nat. Mater..

[175] So C.R., Hayamizu Y., Yazici H., Gresswell C., Khatayevich D., Tamerler C., Sarikaya M. (2012). Controlling Self-Assembly of Engineered Peptides on Graphite by Rational Mutation. ACS Nano.

[176] Peelle B.R., Krauland E.M., Wittrup K.D., Belcher A.M. (2005). Design Criteria for Engineering Inorganic Material- Specific Peptides. Langmuir.

[177] Hnilova M., Oren E.E., Seker U.O.S., Wilson B.R., Collino S., Evans J.S., Tamerler C., Sarikaya M. (2008). Effect of Molecular Conformations on the Adsorption Behavior of Gold-Binding Peptides. Langmuir.

[178] Korkmaz Zirpel N., Arslan T., Lee H. (2015). Engineering filamentous bacteriophages for enhanced gold binding and metallization properties. J. Colloid Interface Sci..

[179] Tamerler C., Kacar T., Sahin D., Fong H., Sarikaya M. (2007). Genetically engineered polypeptides for inorganics: A utility in biological materials science and engineering. Mater. Sci. Eng. C.

[180] Gaskin D.J.H., Starck K., Vulfson E.N. (2000). Identification of inorganic crystal-specific sequences using phage display combinatorial library of short peptides: A feasibility study. Biotechnol. Lett..

[181] Sarikaya M., Tamerler C., Schwartz D.T., Baneyx F.O. (2004). Materials assembly and formation using engineered polypeptides. Annu. Rev. Mater. Res..

[182] Estephan E., Saab M.-B., Larroque C., Martin M., Olsson F., Lourdudoss S., Gergely C. (2009). Peptides for functionalization of InP semiconductors. J. Colloid Interface Sci..

[183] Sano K.-I., Sasaki H., Shiba K. (2005). Specificity and Biomineralization Activities of Ti-Binding Peptide-1 (TBP-1). Langmuir.

[184] Wang S., Humphreys E.S., Chung S.-Y., Delduco D.F., Lustig S.R., Wang H., Parker K.N., Rizzo N.W., Subramoney S., Chiang Y.-M. (2003). Peptides with selective affinity for carbon nanotubes. Nat. Mater..

[185] Chen H., Su X., Neoh K.-G., Choe W.-S. (2006). QCM-D Analysis of Binding Mechanism of Phage Particles Displaying a Constrained Heptapeptide with Specific Affinity to SiO2 and TiO_2_. Anal. Chem..

[186] Huang Y., Chiang C.-Y., Lee S.K., Gao Y., Hu E.L., Yoreo J.D., Belcher A.M. (2005). Programmable Assembly of Nanoarchitectures Using Genetically Engineered Viruses. Nano Lett..

[187] Cui Y., Kim S.N., Jones S.E., Wissler L.L., Naik R.R., McAlpine M.C. (2010). Chemical Functionalization of Graphene Enabled by Phage Displayed Peptides. Nano Lett..

[188] Galloway J.M., Staniland S.S. (2012). Protein and peptide biotemplated metal and metal oxide nanoparticles and their patterning onto surfaces. J. Mater. Chem..

[189] Mao C., Solis D.J., Reiss B.D., Kottmann S.T., Sweeney R.Y., Hayhurst A., Georgiou G., Iverson B., Belcher A.M. (2004). Virus-Based Toolkit for the Directed Synthesis of Magnetic and Semiconducting Nanowires. Science.

[190] Nam Y.S., Park H., Magyar A.P., Yun D.S., Pollom T.S., Belcher A.M. (2012). Virus-templated iridium oxide-gold hybrid nanowires for electrochromic application. Nanoscale.

[191] Mao C., Flynn C.E., Hayhurst A., Sweeney R., Qi J., Georgiou G., Iverson B., Belcher A.M. (2003). Viral assembly of oriented quantum dot nanowires. Proc. Natl. Acad. Sci. USA.

[192] Flynn C.E., Mao C., Hayhurst A., Williams J.L., Georgiou G., Iverson B., Belcher A.M. (2003). Synthesis and organization of nanoscale II-VI semiconductor materials using evolved peptide specificity and viral capsid assembly. J. Mater. Chem..

[193] Reiss B.D., Mao C., Solis D.J., Ryan K.S., Thomson T., Belcher A.M. (2004). Biological Routes to Metal Alloy Ferromagnetic Nanostructures. Nano Lett..

[194] Loo L., Guenther R.H., Basnayake V.R., Lommel S.A., Franzen S. (2006). Controlled Encapsidation of Gold Nanoparticles by a Viral Protein Shell. J. Am. Chem. Soc..

[195] Huang X., Bronstein L.M., Retrum J., Dufort C., Tsvetkova I., Aniagyei S., Stein B., Stucky G., McKenna B., Remmes N. (2007). Self-Assembled Virus-like Particles with Magnetic Cores. Nano Lett..

[196] Minten I.J., Hendriks L.J.A., Nolte R.J.M., Cornelissen J.J.L.M. (2009). Controlled Encapsulation of Multiple Proteins in Virus Capsids. J. Am. Chem. Soc..

[197] Wen A.M., Shukla S., Saxena P., Aljabali A.A.A., Yildiz I., Dey S., Mealy J.E., Yang A.C., Evans D.J., Lomonossoff G.P. (2012). Interior Engineering of a Viral Nanoparticle and Its Tumor Homing Properties. Biomacromolecules.

[198] Loo L., Guenther R.H., Lommel S.A., Franzen S. (2008). Infusion of dye molecules into Red clover necrotic mosaic virus. Chem. Commun..

[199] Ashley C.E., Carnes E.C., Phillips G.K., Durfee P.N., Buley M.D., Lino C.A., Padilla D.P., Phillips B., Carter M.B., Willman C.L. (2011). Cell-Specific Delivery of Diverse Cargos by Bacteriophage MS2 Virus-like Particles. ACS Nano.

[200] Wu M., Sherwin T., Brown W.L., Stockley P.G. (2005). Delivery of antisense oligonucleotides to leukemia cells by RNA bacteriophage capsids. Nanomed. Nanotechnol. Biol. Med..

[201] Wei B., Wei Y., Zhang K., Wang J., Xu R., Zhan S., Lin G., Wang W., Liu M., Wang L. (2009). Development of an antisense RNA delivery system using conjugates of the MS2 bacteriophage capsids and HIV-1 TAT cell penetrating peptide. Biomed. Pharmacother..

[202] Glasgow J.E., Capehart S.L., Francis M.B., Tullman-Ercek D. (2012). Osmolyte-Mediated Encapsulation of Proteins inside MS2 Viral Capsids. ACS Nano.

[203] Capehart S.L., Coyle M.P., Glasgow J.E., Francis M.B. (2013). Controlled Integration of Gold Nanoparticles and Organic Fluorophores Using Synthetically Modified MS2 Viral Capsids. J. Am. Chem. Soc..

[204] Liu C., Chung S.-H., Jin Q., Sutton A., Yan F., Hoffmann A., Kay B.K., Bader S.D., Makowski L., Chen L. (2006). Magnetic viruses via nano-capsid templates. J. Magn. Magn. Mater..

[205] Edgar R., McKinstry M., Hwang J., Oppenheim A.B., Fekete R.A., Giulian G., Merril C., Nagashima K., Adhya S. (2006). High-sensitivity bacterial detection using biotin-tagged phage and quantum-dot nanocomplexes. Proc. Natl. Acad. Sci. USA.

[206] Oh M.H., Yu J.H., Kim I., Nam Y.S. (2015). Genetically Programmed Clusters of Gold Nanoparticles for Cancer Cell-Targeted Photothermal Therapy. ACS Appl. Mater. Interfaces.

[207] Aanei I.L., Huynh T., Seo Y., Francis M.B. (2018). Vascular Cell Adhesion Molecule-Targeted MS2 Viral Capsids for the Detection of Early-Stage Atherosclerotic Plaques. Bioconj. Chem..

[208] Farkas M.E., Aanei I.L., Behrens C.R., Tong G.J., Murphy S.T., O’Neil J.P., Francis M.B. (2013). PET Imaging and Biodistribution of Chemically Modified Bacteriophage MS2. Mol. Pharm..

[209] Anderson E.A., Isaacman S., Peabody D.S., Wang E.Y., Canary J.W., Kirshenbaum K. (2006). Viral Nanoparticles Donning a Paramagnetic Coat:  Conjugation of MRI Contrast Agents to the MS2 Capsid. Nano Lett..

[210] Ghosh D., Bagley A.F., Na Y.J., Birrer M.J., Bhatia S.N., Belcher A.M. (2014). Deep, noninvasive imaging and surgical guidance of submillimeter tumors using targeted M13-stabilized single-walled carbon nanotubes. Proc. Natl. Acad. Sci. USA.

[211] Ghosh D., Lee Y., Thomas S., Kohli A.G., Yun D.S., Belcher A.M., Kelly K.A. (2012). M13-templated magnetic nanoparticles for targeted in vivo imaging of prostate cancer. Nat. Nano.

[212] Xu Z., Sun H., Gao F., Hou L., Li N. (2012). Synthesis and magnetic property of T4 virus-supported gold-coated iron ternary nanocomposite. J. Nanopart. Res..

[213] Hou L., Gao F., Li N. (2010). T4 Virus-Based Toolkit for the Direct Synthesis and 3D Organization of Metal Quantum Particles. Chem. Eur. J..

[214] Hou L., Tong D., Jiang Y., Gao F. (2014). Synthesis and organization of platinum nanoparticles and nanoshells on a native virus bioscaffold. Nano.

[215] Frenkel D., Solomon B. (2002). Filamentous phage as vector-mediated antibody delivery to the brain. Proc. Natl. Acad. Sci. USA.

[216] Bar H., Yacoby I., Benhar I. (2008). Killing cancer cells by targeted drug-carrying phage nanomedicines. BMC Biotechnol..

[217] Suthiwangcharoen N., Li T., Li K., Thompson P., You S., Wang Q. (2011). M13 bacteriophage-polymer nanoassemblies as drug delivery vehicles. Nano Res..

[218] Ren Y., Wong S.M., Lim L.-Y. (2007). Folic Acid-Conjugated Protein Cages of a Plant Virus:  A Novel Delivery Platform for Doxorubicin. Bioconj. Chem..

[219] Pitek A.S., Hu H., Shukla S., Steinmetz N.F. (2018). Cancer Theranostic Applications of Albumin-Coated Tobacco Mosaic Virus Nanoparticles. ACS Appl. Mater. Interfaces.

[220] Le D.H.T., Lee K.L., Shukla S., Commandeur U., Steinmetz N.F. (2017). Potato virus X, a filamentous plant viral nanoparticle for doxorubicin delivery in cancer therapy. Nanoscale.

[221] Alemzadeh E., Izadpanah K., Ahmadi F. (2017). Generation of recombinant protein shells of Johnson grass chlorotic stripe mosaic virus in tobacco plants and their use as drug carrier. J. Virol. Methods.

[222] Alemzadeh E., Dehshahri A., Dehghanian A.R., Afsharifar A., Behjatnia A.A., Izadpanah K., Ahmadi F. (2019). Enhanced anti-tumor efficacy and reduced cardiotoxicity of doxorubicin delivered in a novel plant virus nanoparticle. Colloids Surf. B Biointerfaces.

[223] Kumar K., Kumar Doddi S., Kalle Arunasree M., Paik P. (2015). CPMV-induced synthesis of hollow mesoporous SiO2 nanocapsules with excellent performance in drug delivery. Dalton Trans..

[224] Yildiz I., Lee K.L., Chen K., Shukla S., Steinmetz N.F. (2013). Infusion of imaging and therapeutic molecules into the plant virus-based carrier cowpea mosaic virus: Cargo-loading and delivery. J. Control. Release.

[225] Liu X., Liu B., Gao S., Wang Z., Tian Y., Wu M., Jiang S., Niu Z. (2017). Glyco-decorated tobacco mosaic virus as a vector for cisplatin delivery. J. Mater. Chem. B.

[226] Franke C.E., Czapar A.E., Patel R.B., Steinmetz N.F. (2018). Tobacco Mosaic Virus-Delivered Cisplatin Restores Efficacy in Platinum-Resistant Ovarian Cancer Cells. Mol. Pharm..

[227] DePorter S.M., McNaughton B.R. (2014). Engineered M13 Bacteriophage Nanocarriers for Intracellular Delivery of Exogenous Proteins to Human Prostate Cancer Cells. Bioconj. Chem..

[228] Sánchez-Sánchez L., Cadena-Nava R.D., Palomares L.A., Ruiz-Garcia J., Koay M.S.T., Cornelissen J.J.M.T., Vazquez-Duhalt R. (2014). Chemotherapy pro-drug activation by biocatalytic virus-like nanoparticles containing cytochrome P450. Enzym. Microb. Technol..

[229] Hoskins J.M., Carey L.A., McLeod H.L. (2009). *CYP2D6* and tamoxifen: DNA matters in breast cancer. Nat. Rev. Cancer.

[230] Yacoby I., Shamis M., Bar H., Shabat D., Benhar I. (2006). Targeting Antibacterial Agents by Using Drug-Carrying Filamentous Bacteriophages. Antimicrob. Agents Chemother..

[231] Brasch M., de la Escosura A., Ma Y., Uetrecht C., Heck A.J.R., Torres T., Cornelissen J.J.L.M. (2011). Encapsulation of Phthalocyanine Supramolecular Stacks into Virus-like Particles. J. Am. Chem. Soc..

[232] Lee K.L., Carpenter B.L., Wen A.M., Ghiladi R.A., Steinmetz N.F. (2016). High Aspect Ratio Nanotubes Formed by Tobacco Mosaic Virus for Delivery of Photodynamic Agents Targeting Melanoma. ACS Biomater. Sci. Eng..

[233] Chariou P.L., Wang L., Desai C., Park J., Robbins L.K., von Recum H.A., Ghiladi R.A., Steinmetz N.F. (2019). Let There Be Light: Targeted Photodynamic Therapy Using High Aspect Ratio Plant Viral Nanoparticles. Macromol. Biosci..

[234] Ngweniform P., Abbineni G., Cao B., Mao C. (2009). Self-Assembly of Drug-Loaded Liposomes on Genetically Engineered Target-Recognizing M13 Phage: A Novel Nanocarrier for Targeted Drug Delivery. Small.

[235] Sawada T., Yanagimachi M., Serizawa T. (2017). Controlled release of antibody proteins from liquid crystalline hydrogels composed of genetically engineered filamentous viruses. Mater. Chem. Front..

[236] Cao J., Liu S., Chen Y., Shi L., Zhang Z. (2014). Synthesis of end-functionalized boronic acid containing copolymers and their bioconjugates with rod-like viruses for multiple responsive hydrogels. Polym. Chem..

[237] Zhi X., Zheng C., Xiong J., Li J., Zhao C., Shi L., Zhang Z. (2018). Nanofilamentous Virus-Based Dynamic Hydrogels with Tunable Internal Structures, Injectability, Self-Healing, and Sugar Responsiveness at Physiological pH. Langmuir.

[238] Honarbakhsh S., Guenther R.H., Willoughby J.A., Lommel S.A., Pourdeyhimi B. (2013). Polymeric Systems Incorporating Plant Viral Nanoparticles for Tailored Release of Therapeutics. Adv. Healthc. Mater..

[239] Tian Y., Wu M., Liu X., Liu Z., Zhou Q., Niu Z., Huang Y. (2015). Probing the Endocytic Pathways of the Filamentous Bacteriophage in Live Cells Using Ratiometric pH Fluorescent Indicator. Adv. Healthc. Mater..

[240] Koudelka K.J., Destito G., Plummer E.M., Trauger S.A., Siuzdak G., Manchester M. (2009). Endothelial targeting of cowpea mosaic virus (CPMV) via surface vimentin. PLoS Pathog..

[241] Srivastava A.S., Kaido T., Carrier E. (2004). Immunological factors that affect the in vivo fate of T7 phage in the mouse. J. Virol. Methods.

[242] Aanei I.L., ElSohly A.M., Farkas M.E., Netirojjanakul C., Regan M., Taylor Murphy S., O’Neil J.P., Seo Y., Francis M.B. (2016). Biodistribution of Antibody-MS2 Viral Capsid Conjugates in Breast Cancer Models. Mol. Pharm..

[243] Wu M., Shi J., Fan D., Zhou Q., Wang F., Niu Z., Huang Y. (2013). Biobehavior in Normal and Tumor-Bearing Mice of Tobacco Mosaic Virus. Biomacromolecules.

[244] Petrenko V.A. (2017). Autonomous self-navigating drug-delivery vehicles: From science fiction to reality. Ther. Deliv..

[245] Le D.H.T., Méndez-López E., Wang C., Commandeur U., Aranda M.A., Steinmetz N.F. (2019). Biodistribution of Filamentous Plant Virus Nanoparticles: Pepino Mosaic Virus versus Potato Virus X. Biomacromolecules.

[246] Yip Y.L., Hawkins N.J., Smith G., Ward R.L. (1999). Biodistribution of filamentous phage-Fab in nude mice. J. Immunol. Methods.

[247] Wu Z., Chen K., Yildiz I., Dirksen A., Fischer R., Dawson P.E., Steinmetz N.F. (2012). Development of viral nanoparticles for efficient intracellular delivery. Nanoscale.

[248] Raja K.S., Wang Q., Gonzalez M.J., Manchester M., Johnson J.E., Finn M.G. (2003). Hybrid Virus−Polymer Materials. 1. Synthesis and Properties of PEG-Decorated Cowpea Mosaic Virus. Biomacromolecules.

[249] Steinmetz N.F., Manchester M. (2009). PEGylated Viral Nanoparticles for Biomedicine: The Impact of PEG Chain Length on VNP Cell Interactions In Vitro and Ex Vivo. Biomacromolecules.

[250] Bruckman M.A., Randolph L.N., VanMeter A., Hern S., Shoffstall A.J., Taurog R.E., Steinmetz N.F. (2014). Biodistribution, pharmacokinetics, and blood compatibility of native and PEGylated tobacco mosaic virus nano-rods and -spheres in mice. Virology.

[251] Bludau H., Czapar A.E., Pitek A.S., Shukla S., Jordan R., Steinmetz N.F. (2017). POxylation as an alternative stealth coating for biomedical applications. Eur. Polym. J..

[252] Pitek A.S., Jameson S.A., Veliz F.A., Shukla S., Steinmetz N.F. (2016). Serum albumin ‘camouflage’ of plant virus based nanoparticles prevents their antibody recognition and enhances pharmacokinetics. Biomaterials.

[253] Krag D.N., Shukla G.S., Shen G.-P., Pero S., Ashikaga T., Fuller S., Weaver D.L., Burdette-Radoux S., Thomas C. (2006). Selection of Tumor-binding Ligands in Cancer Patients with Phage Display Libraries. Cancer Res..

[254] Staquicini F.I., Cardó-Vila M., Kolonin M.G., Trepel M., Edwards J.K., Nunes D.N., Sergeeva A., Efstathiou E., Sun J., Almeida N.F. (2011). Vascular ligand-receptor mapping by direct combinatorial selection in cancer patients. Proc. Natl. Acad. Sci. USA.

[255] Schubbert R., Renz D., Schmitz B., Doerfler W. (1997). Foreign (M13) DNA ingested by mice reaches peripheral leukocytes, spleen, and liver via the intestinal wall mucosa and can be covalently linked to mouse DNA. Proc. Natl. Acad. Sci. USA.

